# Increased adipose catecholamine levels and protection from obesity with loss of Allograft Inflammatory Factor-1

**DOI:** 10.1038/s41467-022-35683-7

**Published:** 2023-01-03

**Authors:** Prameladevi Chinnasamy, Isabel Casimiro, Dario F. Riascos-Bernal, Shreeganesh Venkatesh, Dippal Parikh, Alishba Maira, Aparna Srinivasan, Wei Zheng, Elena Tarabra, Haihong Zong, Smitha Jayakumar, Venkatesh Jeganathan, Kith Pradan, Jose O. Aleman, Rajat Singh, Sayan Nandi, Jeffrey E. Pessin, Nicholas E. S. Sibinga

**Affiliations:** 1 Department of Medicine (Cardiology), Albert Einstein College of Medicine, Bronx, NY USA; 2 Department of Developmental and Molecular Biology, Albert Einstein College of Medicine, Bronx, NY USA; 3 Wilf Family Cardiovascular Research Institute, Albert Einstein College of Medicine, Bronx, NY USA; 4 Albert Einstein Cancer Center, Albert Einstein College of Medicine, Bronx, NY USA; 5 Department of Molecular Pharmacology, Albert Einstein College of Medicine, Bronx, NY USA; 6 Department of Medicine (Endocrinology, Albert Einstein College of Medicine), Bronx, NY USA; 7 Einstein-Mount Sinai Diabetes Research Center and Fleischer Institute of Diabetes and Metabolism, Albert Einstein College of Medicine, Bronx, NY USA; 8 Department of Epidemiology and Population Health, Albert Einstein College of Medicine, Bronx New York, USA; 9 Department of Medicine (Endocrinology), New York University Langone Health, New York, NY USA

**Keywords:** Obesity, Fat metabolism

## Abstract

Recent studies implicate macrophages in regulation of thermogenic, sympathetic neuron-mediated norepinephrine (NE) signaling in adipose tissues, but understanding of such non-classical macrophage activities is incomplete. Here we show that male mice lacking the allograft inflammatory factor-1 (AIF1) protein resist high fat diet (HFD)-induced obesity and hyperglycemia. We link this phenotype to higher adipose NE levels that stem from decreased monoamine oxidase A (MAOA) expression and NE clearance by AIF1-deficient macrophages, and find through reciprocal bone marrow transplantation that donor *Aif1*^*-/-*^ vs WT genotype confers the obesity phenotype in mice. Interestingly, human sequence variants near the *AIF1* locus associate with obesity and diabetes; in adipose samples from participants with obesity, we observe direct correlation of *AIF1* and *MAOA* transcript levels. These findings identify AIF1 as a regulator of MAOA expression in macrophages and catecholamine activity in adipose tissues – limiting energy expenditure and promoting energy storage – and suggest how it might contribute to human obesity.

## Abstract (web summary)

Macrophages have been reported to regulate thermogenic, sympathetic neuron-mediated norepinephrine signaling in adipose tissues. Here the authors report that male mice lacking the allograft inflammatory factor-1 protein are resistant to diet-induced obesity, in association with higher adipose norepinephrine levels and lower expression of the norepinephrine catabolic enzyme monoamine oxidase A.

## Introduction

Obesity and an array of multiple obesity-associated diseases—including type 2 diabetes, insulin resistance, cardiovascular disease, and cancer—have been increasing in prevalence throughout the world^1–3^, so the development of new therapies that can mitigate obesity is a high priority for contemporary biomedicine. Thermogenic activity in brown and white adipose tissues promotes energy expenditure and thus could be useful in such strategies^4^.

Non-classical functions of tissue-resident macrophages contribute to energy homeostasis, including control of thermogenesis in brown/beige fat and insulin sensitivity in WAT^5^. On the other hand, in obesity-induced systemic and adipose tissue inflammation, macrophages are key drivers in adipose tissue dysfunction that contributes to the development of metabolic syndrome^6^. Despite controversies regarding the relationship between macrophages and NE metabolism^7,8^, recent reports suggest that CD45^+^;F4/80^+^ adipose tissue macrophages (ATMs) regulate thermogenic activities through effects on sympathetic neuron-mediated NE signaling^9–13^. However, molecular mechanisms linking ATM functions to NE signaling and their significance in obesity and insulin resistance are not fully understood.

Allograft inflammatory factor-1, a 17 kDa protein preferentially expressed in myeloid cell lineages, may contribute to the development of allo- and autoimmune diseases^14–19^. Studies in human populations link sequence variants near the *AIF1* locus with obesity and diabetes^20,21^, but a potential role for AIF1 in the pathogenesis of these conditions has not been investigated.

Here, we show that loss of AIF1 protects against diet-induced obesity, glucose intolerance, and insulin resistance. These findings are accompanied by increased NE levels and β-adrenergic receptor (AR) signaling in BAT and inguinal WAT (iWAT), and correlate with higher core energy expenditure at low (10 °C), ambient (22 °C), and thermoneutral (30 °C) temperatures. Mechanistically, we observe that AIF1 promotes NE clearance by supporting the expression of MAOA in ATMs. In addition, we provide evidence linking these findings to human obesity, in that *AIF1* expression correlates positively with *MAOA* and *ALDH1L2* levels in adipose tissues from individuals with weight excess or obesity. Collectively, our findings elucidate an AIF1–MAOA axis that affects NE catabolism in macrophages and suggest that it may contribute to obesity and associated insulin resistance and glucose intolerance.

## Results

### AIF1 loss limits HFD-induced obesity and insulin resistance

**Fig. 1:**
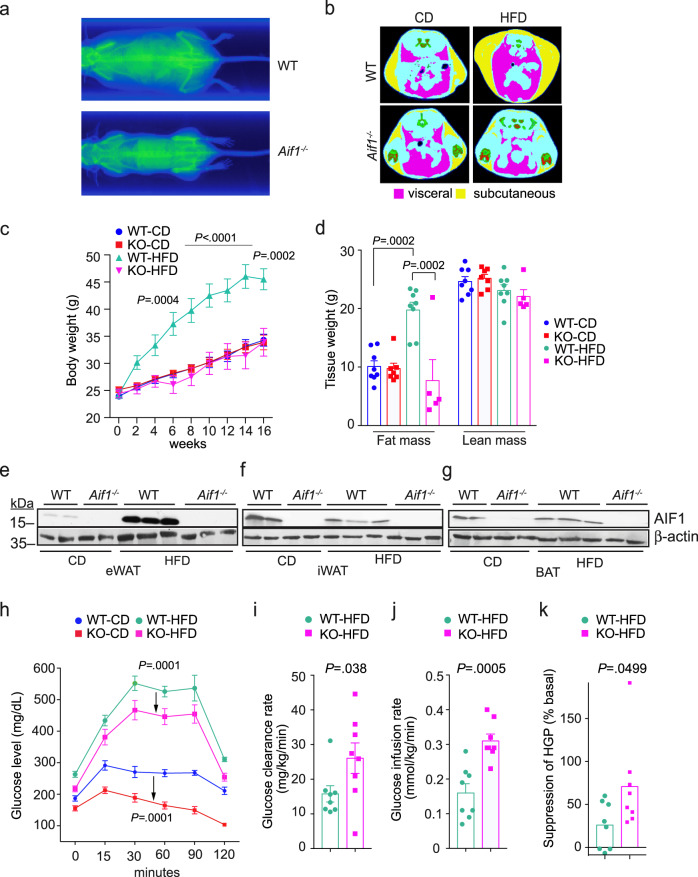
Loss of AIF1 prevents obesity and insulin resistance induced by HFD. WT and *Aif1*^*−/−*^ (KO) mice were assigned to chow (CD) or high-fat diet (HFD) starting at 8 weeks of age. **a**, **b** Computerized tomographic scans were performed after 16 weeks on diet. **a** Whole body projections show skeletal and soft tissue signals. **b** False-colored cross-sectional samples indicate visceral (pink) and subcutaneous (yellow) adipose tissues, plus lean (light blue) and skeletal (green) elements. **c** Body weight measured at the indicated time points after starting on CD or HFD (*n*: WT-CD, 9; KO-CD, 6; WT-HFD, 9; KO-HFD, 9). **d** Fat and lean masses measured by magnetic resonance imaging after 16 weeks on diet (*n*: WT-CD, 8; KO-CD, 7; WT-HFD, 8; KO-HFD, 5). **e**–**g** AIF1 expression after 16 weeks on diet assessed by Western blot in eWAT (**e**), iWAT (**f**), and BAT (**g**). The experiment was repeated 3 times with similar results. **h**–**k** Insulin resistance in WT and *Aif1*^*−/−*^ mice after 16 weeks of CD or HFD. **h** Glucose tolerance test (n: WT-CD, 5; KO-CD, 5; WT-HFD, 4; KO-HFD, 5). Arrows indicate the effect of loss of AIF1 for a given diet. **i**–**k** Euglycemic, hyperinsulinemic clamp studies, showing glucose clearance rate (**i**), glucose infusion rate (**j**), and hepatic glucose production (HGP) (n: WT-HFD, 8; KO-HFD, 8) (**k**). Data are presented as mean ± s.e.m. Differences were evaluated by two-way ANOVA followed by Sidak’s multiple comparison test (**c**), Tukey’s multiple comparison test (**d**, **h**) and for **i**–**k**, two-sided Mann–Whitney *U*-test. *n* = the number of biologically independent animals. Source data are provided as a Source Data file.

Mice lacking AIF1 appear grossly normal and show no obvious behavioral phenotype^14^. To evaluate possible links between the *Aif1* locus and obesity, we compared the responses of WT and *Aif1*^*−/−*^ mice to HFD feeding over 16 weeks. WT mice developed characteristic features of obesity that were not seen in *Aif1*^*−/−*^ mice, and CT scanning revealed marked expansion of both subcutaneous and visceral adipose depots in HFD-fed WT mice also not found in HFD-fed *Aif1*^*−/−*^ mice, or in mice of either genotype on standard chow diet (CD) (Fig. 1a, b). Similarly, corresponding weight curves climbed steeply in HFD-fed WT mice, while curves of *Aif1*^*−/−*^ mice on HFD were flatter and overlapped those of CD-fed WT or *Aif1*^*−/−*^ mice (Fig. 1c). Evaluation of fat and lean tissues by magnetic resonance imaging (MRI) indicated that this protection in HFD-fed *Aif1*^*−/−*^ mice reflected a lack of increase in fat mass, with no differences in lean mass across all groups (Fig. 1d). Evaluation of AIF1 expression in white and brown adipose tissue (WAT and BAT, respectively) documented its presence in epididymal WAT (eWAT), inguinal WAT (iWAT), and BAT, with HFD-induced increase only in eWAT (Fig. 1e–g). This increase in AIF1 may reflect the accumulation of macrophages and crown-like structures^22^ apparent in hematoxylin–eosin stains of WT eWAT from HFD-fed mice (Supplementary Fig. 1a). Loss of AIF1 appeared to limit adipocyte size in iWAT and lipid accumulation in BAT, with greater effects with HFD than CD (Supplementary Fig. 1b, c).

We then performed glucose tolerance testing to evaluate if protection from weight gain and eWAT inflammation due to AIF1 loss also affected glucose handling. With HFD feeding, glucose levels in both WT and *Aif1*^*−/−*^ mice were higher than with CD; however, mice lacking AIF1 and fed with either CD or HFD showed relative protection against hyperglycemia (Fig. 1h). Further evaluation using euglycemic, hyperinsulinemic clamp methods indicated higher glucose clearance and suppression of hepatic glucose production, consistent with improved insulin sensitivity and less hepatosteatosis in *Aif1*^*−/−*^ mice (Fig. 1i–k; Supplementary Fig. 1d, e).

### Increased energy expenditure in *Aif1*^*−/−*^ vs. WT mice

**Fig. 2:**
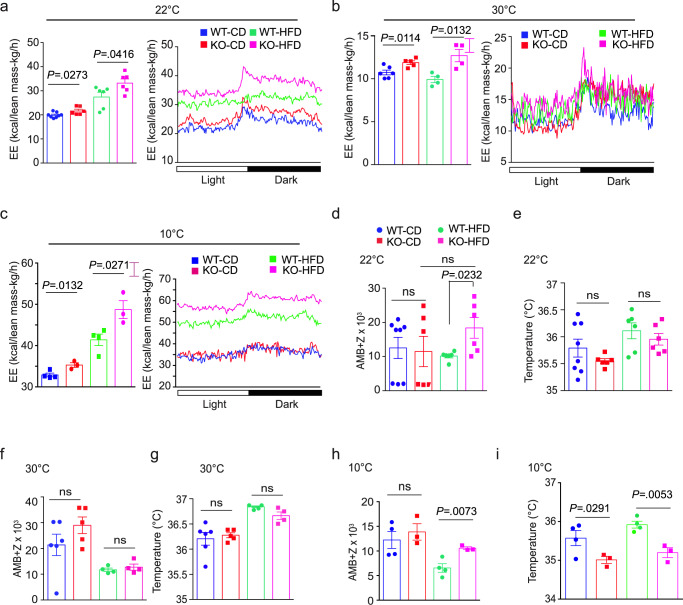
Mice lacking AIF1 expend more energy in both ambient and thermoneutral conditions, independent of changes in activity or temperature. Metabolic characteristics of AIF1-deficient and control mice fed with CD or HFD for 16 weeks were measured using metabolic cages at ambient temperature (22 °C), at thermoneutrality (30 °C), or at reduced temperature (10 °C). **a** Energy expenditure (EE) at 22 °C, showing mean values collected over 4 days (left panel) and over a 24 h light-dark cycle (right panel) (*n*: WT-CD, 8; KO-CD, 6; WT-HFD, 6; KO-HFD, 6). **b**, **c** Similar assessments, but with mice housed at 30 °C (**b**) (*n*: WT-CD, 6; KO-CD, 5; WT-HFD, 4; KO-HFD, 4) or 10 °C (**c**) (*n*: WT-CD, 4; KO-CD, 3; WT-HFD, 4; KO-HFD, 3). **d** Physical activity measured by beam breaks, or **e** temperature, showing mean values collected over 4 days at 22 °C (*n*: WT-CD, 8; KO-CD, 6; WT-HFD, 6; KO-HFD, 6). **f** Physical activity measured by beam breaks, or **g**, temperature, showing mean values collected over 4 days at 30 °C (*n*: WT-CD, 6; KO-CD, 5; WT-HFD, 4; KO-HFD, 4). **h** Physical activity measured by beam breaks, or **i** temperature, showing mean values collected over 5 days at 10 °C (*n*: WT-CD, 4; KO-CD, 3; WT-HFD, 4; KO-HFD, 3). Data are presented as mean ± s.e.m.; significance is assessed by unpaired two-sided Student’s *t*-test. Data are representative of three similar experiments. *n* = number of biologically independent animals. Source data are provided as a Source Data file.

To understand the nature of this resistance to obesity and hyperglycemia, we performed indirect calorimetry and activity analyses. Reduced food intake could limit weight gain and insulin resistance, but on either CD or HFD, there was no significant difference in intake (Supplementary Fig. 2a). In each of three different environmental temperatures (ambient (22 °C), thermoneutral (30 °C), or cold (10 °C)), *Aif1*^*−/−*^ mice showed greater energy expenditure, regardless of diet (Fig. 2a–c). Oxygen consumption and carbon dioxide production were also higher in these mice in all conditions except CD at 10 °C, which showed a similar trend (Supplementary Fig. 2b–f). These changes were consistent throughout the diurnal cycle. Increased energy expenditure was not clearly explained by greater physical activity, as ambulatory +*Z* scores of WT and *Aif1*^*−/−*^ mice on CD (Fig. 2d, f, h) were similar at each temperature. WT mice on HFD scored relatively lower at 10 and 22 °C, perhaps secondary to their obese state (Fig. 2d, h), but this is unlikely to contribute significantly to differences in energy expenditure^23^. Despite the increase in energy expenditure, *Aif1*^*−/−*^ mice had body temperatures similar to WT when housed at 22 or 30 °C (Fig. 2e, g), and mildly lower body temperature when housed at 10 °C (Fig. 2i).

### AIF1 deficiency limits HFD-induced BAT and iWAT expansion

Having excluded differences in food intake, physical activity, and body temperature as explanations for the increased energy expenditure and obesity resistance in *Aif1*^*−/−*^ mice, we examined adipose tissues. As suggested by imaging data (Fig. 1), HFD induced a marked increase in the mass of iWAT and BAT depots from WT, but not *Aif1*^*−/−*^ mice (Supplementary Fig. 3a–d). In addition, BAT from *Aif1*^*−/−*^ mice appeared darker in color (Supplementary Fig. 3b), consistent with lower fat content (Supplementary Fig. 1c). Liver and skeletal muscle masses were not affected by diet or genotype (Supplementary Fig. 3e–h).

### AIF1 deficiency increases BAT metabolic activity

Next, we sought to understand how AIF1 loss might affect adipose tissue phenotype. Higher expression of candidate genes associated with thermogenesis—including uncoupling protein-1 (*Ucp1*) and iodothyronine deiodinase 2 (*DiO2*)^24,25^ in *Aif1*^*−/−*^ BAT, and cell death-inducing DNA fragmentation factor alpha-like effector A (*Cidea*) and transmembrane protein 26 (*Tmem26*) in *Aif1*^*−/−*^ iWAT^26–28^—suggested increased metabolic activity in adipose tissues of AIF1-deficient mice on both CD and HFD (Supplementary Fig. 4a, Supplementary Table 1). UCP1 protein expression in *Aif1*^*−/−*^ BAT and iWAT tissues was also increased (Supplementary Fig. 4b–d). The levels of mitochondrial respiratory complexes I–V in BAT and iWAT of *Aif1*^*−/−*^ mice (assessed by quantifying proteins representative for each complex; Supplementary Fig. 5a, b) were not different from those of WT, suggesting that the higher levels of UCP1 were not due to an increase in overall mitochondrial content in the *Aif1*^*−/−*^ tissues.

We then evaluated metabolic function directly using high-resolution tissue respirometry^29,30^. Compared to WT BAT, the oxygen consumption rate (OCR) of *Aif1*^*−/−*^ BAT was higher, and this increase was prevented by treatment with propranolol, a non-selective β-adrenergic antagonist (Supplementary Fig. 5c, d). The OCRs of both WT and *Aif1*^*−/−*^ iWAT were also limited by propranolol, but overall levels did not differ according to genotype, perhaps reflecting the relatively lower fraction of thermogenic cells in iWAT tissue (Supplementary Fig. 5d). The propranolol sensitivity of the observed increase in OCR, together with prior studies of different β-adrenergic receptor subtypes in BAT^31^, suggested that the increased metabolic activity due to AIF1 deficiency was mediated primarily through the β_3_-adrenergic receptor. Signaling downstream of the β_3_-adrenergic receptor is known to increase cyclic AMP and protein kinase A activity^32^, and consistent with activation of this canonical pathway in adipose tissues lacking AIF1, we found increased phosphorylation of hormone-sensitive lipase (HSL) (Supplementary Fig. 5e–g).

### Lack of AIF1 affects adipose tissue neurotransmitter levels

**Fig. 3:**
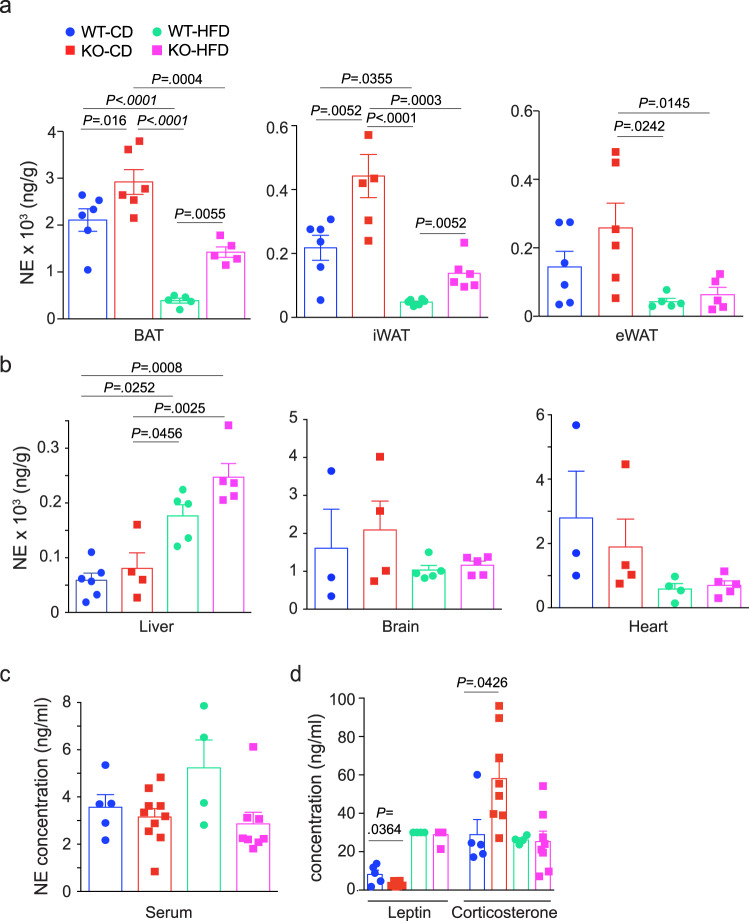
The increase in NE levels with AIF1 deficiency is restricted to adipose tissues. WT and AIF1-deficient (KO) mice were fed with CD or HFD. After 16 weeks of diet, NE levels were measured by LC/MS. **a**–**c** NE levels in adipose depots (*n*: BAT and eWAT: WT-CD, 6; KO-CD, 6; WT-HFD, 5; KO-HFD, 5; iWAT: *n* = 6 for all groups), (**a**), in the liver (*n*: WT-CD, 6; KO-CD, 4; WT-HFD, 5; KO-HFD, 5); brain (*n*: WT-CD, 3; KO-CD, 4; WT-HFD, 5; KO-HFD, 5); and heart (*n*: WT-CD, 3; KO-CD, 4; WT-HFD, 4; KO-HFD, 5) (**b**), and in serum (n: WT-CD, 5; KO-CD, 10; WT-HFD, 4; KO-HFD, 8) (**c**). **d** Serum leptin and corticosterone concentrations, as measured by ELISA (*n*: WT-CD, 5; KO-CD, 6; WT-HFD, 4; KO-HFD, 8). Data are presented as mean;± s.e.m. Significance was assessed by unpaired two-sided *t*-test (**d**) or one-way ANOVA followed by Tukey’s multiple comparison test (**a**–**c**). *n* = the number of biologically independent animals. Source data are provided as a Source Data file.

BAT thermogenic activity increases in response to NE release from innervating sympathetic fibers^31^, and sensitivity of BAT to this β-adrenergic stimulation can be enhanced by loss or inhibition of serotonin (5-hydroxytryptamine, or 5-HT) signaling in the periphery^33^. To evaluate how these factors might contribute to the metabolic phenotype of AIF1-deficient mice, we checked 5-HT and NE levels in adipose tissues. Levels of 5-HT in adipose tissues, liver, heart, and circulation were not suppressed by AIF1 deficiency, and serum levels of 5-hydroxyindoleacetic acid (5-HIAA), a major 5-HT metabolite, were unchanged (Supplementary Fig. 6a–c, e)—these findings argue against a role for peripheral 5-HT in the obesity resistance phenotype. On the other hand, we found increased NE content in both BAT and iWAT of AIF1-deficient mice fed a CD, while with HFD, the corresponding NE levels decreased in WT mice, but were relatively higher in AIF1-deficient mice (Fig. 3a, Supplementary Fig. 6d). NE levels in liver, heart, and serum were not changed by AIF1 deficiency (Fig. 3b,c) and serum levels of homovanillic acid (HVA), a major catecholamine metabolite, were also not different (Supplementary Fig. 6e). Taken together, these findings suggest that loss of AIF1 increases catecholamine levels discretely in tissues with thermogenic capability.

How this local increase in catecholamines affects adipose tissues is of interest. To look for possible effects on adipocyte phenotype, we isolated stromal-vascular fractions (SVFs) from adipose depots of WT and *Aif1*^*−/−*^ mice, differentiated the cells in vitro to promote brown adipogenesis, and performed respirometry. These studies showed higher ATP-independent OCRs by *Aif1*^*−/−*^ vs. WT adipocytes derived from BAT and iWAT, consistent with an increase in the metabolic capacity of adipocytes lacking AIF1 (Supplementary Fig. 7). In this model of differentiation, cells are treated after harvest with a cocktail of agents (see the “Methods” section) that promote differentiation. Conceivably, the observed increase in ATP-independent (uncoupled) respiration could stem from the effects of higher catecholamine levels prior to harvest that expands the population of (or otherwise predispose) progenitors in AIF1-deficient adipose tissues to browning, or it could stem from catecholamine-independent intrinsic differences in AIF1-deficient progenitors that support the acquisition of brown fate and function in response to the cocktail. From a mechanistic perspective, these findings raise the possibility that the effects of AIF1 deficiency on non-myeloid cells could contribute to higher OCR. Conditional inactivation of AIF1 expression in myeloid vs. adipocyte lineages will be required to address this point.

We also considered if AIF1 deficiency might have similar effects on neurotransmitter levels in the CNS. The central metabolic activities of NE are complex and include both anorexigenic and thermogenic functions^34,35^ that could contribute to obesity resistance. Levels of NE in total brain samples of WT and *Aif1*^*−/−*^ mice were not different, however (Fig. 3b). Central 5-HT signaling is anorexigenic, and in contrast to 5-HT signaling in the periphery, can indirectly increase energy expenditure by stimulating thermogenesis in brown adipose tissue^36,37^. Levels of 5-HT in the brains of HFD-fed mice lacking AIF1 were not, however, significantly higher than in WT mice (Supplementary Fig. 6b). To evaluate possible effects on the hypothalamic–pituitary–adrenal axis^38^, we evaluated circulating leptin and corticosterone levels, and found genotype-dependent changes in mice fed with CD, but not with HFD (Fig. 3d)—like the neurotransmitter studies, these findings did not implicate a CNS-based effect of AIF1 deficiency in the observed resistance to HFD-induced obesity.

### AIF1 deficiency in bone marrow-derived macrophages

**Fig. 4:**
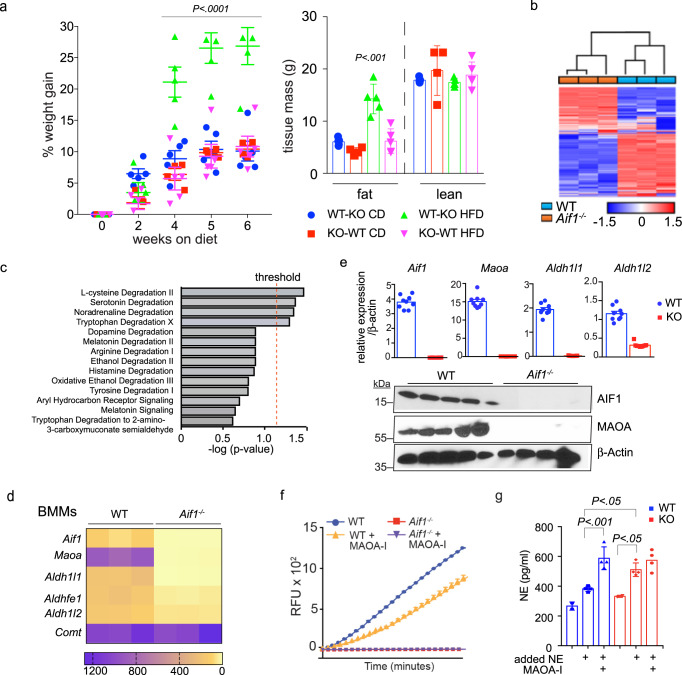
AIF1 expression in bone marrow-derived cells affects obesity resistance. **a** Total bone marrow cells from WT or *Aif1*^*−/−*^ donor mice (age 6–8 weeks) were transferred to irradiated WT or *Aif1*^*−/−*^ recipient mice (age 4–6 weeks). After 6 weeks of recovery, mice were randomly assigned to CD or HFD for 6 weeks and weighed regularly. Key indicates donor-recipient and diet, e.g., WT-KO CD. **a** (left) %weight gain; (right), fat and lean masses, assessed by MRI after 6 weeks on diet. *n*: WT-KO CD, 5; KO-WT CD, 4; WT-KO HFD, 4; KO-WT HFD, 5. Data were evaluated by two-way ANOVA followed by Tukey’s multiple comparison test and presented as mean ± s.e.m. **b**–**d** RNA-seq data obtained from immortalized BMMs generated from male WT and *Aif1*^*−/−*^ mice (*n* = 3 biologically independent animals). **b** Unsupervised hierarchical clustering analysis. **c** Bioamine degradation pathways predicted by GO analysis. **d** heat map representing the expression of key genes in the NE degradation pathway. **e** (upper) expression of *Aif1* and NE catabolism genes (*Maoa, Aldh1/1*, and *Aldh1/2*) in BMMs validated by qRT-PCR (top) (*n*: WT, 9; KO, 8, pooled from 3 independent isolations and presented as mean ± s.e.m.); **e** (lower), protein expression of AIF1 and MAOA in BMMs assessed by immunoblotting, with β-actin shown as a loading control (*n*: WT, 5; KO, 4, pooled from 3 independent cell isolations and representative of 3 similar experiments). **f** MAOA activity measured in WT vs. *Aif1*^*−/−*^ BMMs with or without MAOA inhibitor (MAOA-I (clorgylline, 10 μM)). RFU relative fluorescent units (*n* = 2, graph shows mean ± s.e.m. and is representative of two similar experiments). **g** Catecholamine clearance–NE (2 μM) was added to WT and *Aif1*^*−/−*^ BMMs in culture and cellular NE levels were assessed after 2 h. MAOA-I, clorgylline, 10 μM. *n* = 3 per group for baseline measurement and *n* = 4 per group for other conditions and are representative of 2 similar experiments. Data are presented as mean ± s.e.m. Significance was assessed by one-way ANOVA with Tukey’s multiple comparisons test. Source data are provided as a Source Data file.

Recent studies have identified potential roles for macrophages in adipose tissue NE signaling^12^. While macrophages appear incapable of producing NE^8^, multiple reports indicate that macrophages participate directly or indirectly in the control of NE levels and thereby affect thermogenic^9,11^ or lipolytic^10^ activities in adipocytes; this suggested that AIF1 deficiency might alter adipose catecholamine levels through effects on macrophage function. To address this possibility, we performed reciprocal bone marrow transplantation between WT and *Aif1*^*−/−*^ mice and evaluated weight gain and body composition over the course of 6 weeks on CD or HFD. Notably, *Aif1*^*−/−*^ mice receiving WT bone marrow (WT-KO) on HFD gained significantly more weight than WT-KO mice on CD, or than WT recipients of *Aif1*^*−/−*^ bone marrow (KO-WT) on either CD or HFD (Fig. 4a, Supplementary 8a). As with WT and global *Aif1*^*−/−*^ mice on HFD (Fig. 1), this increase was driven by adipose tissue expansion without a significant change in lean tissue mass (Fig. 4a).

These observations suggested that AIF1 expression in bone marrow-derived cells is important for AIF1-dependent effects on energy storage driven by HFD. To understand how AIF1 deficiency affects relevant cell functions, we performed RNA-seq and compared the transcriptomes of WT and *Aif1*^*−/−*^ bone marrow-derived macrophages (BMMs) (Fig. 4b, c). Pathway analysis identified differences in immune activation and inflammation (Supplementary Data 1, ingenuity pathway analysis of WT vs. *Aif1*^*−/−*^ bone marrow macrophage RNA-seq data), as anticipated based on previous characterizations of AIF1 function. Interestingly, we also found that loss of AIF1 selectively affected macrophage expression of genes associated with monoamine degradation: transcripts encoding key NE degradation pathway components such as monoamine oxidase A (*Maoa*) and aldehyde dehydrogenases (*Aldh1/1, Aldh1/2*, and *AldhFe1*) were markedly decreased, whereas catechol-O-methyl transferase (*Comt*) transcript levels were unchanged (Fig. 4d, e). Consistent with these findings, both MAOA protein and activity were readily detected in WT, but not AIF1-deficient, BMMs (Fig. 4e, f). Moreover, cellular NE levels rose significantly in AIF1-deficient but not WT BMMs challenged by the addition of exogenous NE, suggesting that loss of AIF1 affects the catecholamine clearance ability of these cells (Fig. 4g).

### AIF1 deficiency impairs ATM catecholamine metabolism

**Fig. 5:**
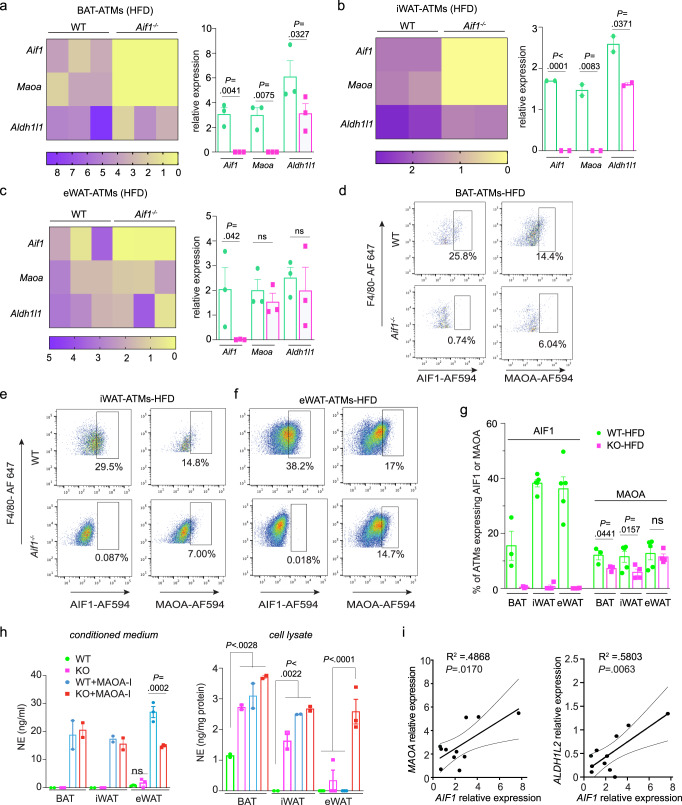
Loss of AIF1 impairs catecholamine catabolic enzyme expression and activity in ATMs. **a**–**c** Expression of key genes in NE degradation, assessed in WT vs. *Aif1*^*−/−*^ in ATMs by qRT-PCR.ATMs were sorted from pooled SVF of BAT (**a**), iWAT (**b**), and eWAT (**c**) obtained from WT or *Aif1*^*−/−*^ mice (*n* = 3 (BAT, eWAT) or 2 (iWAT) independent pools, each pool from 5 mice) after 16 weeks of HFD feeding; heat map (left), bar graph (right). Data pooled from 3 independent experiments **d**–**g** Flow cytometric analysis of MAOA expression in CD45 + CD11b + F4/80+ cells from SVF BAT (*n*: WT, 3; KO, 3) (**d**), iWAT (*n*: WT, 5; KO, 4) (**e**) and eWAT(n: WT, 5; KO, 4) (**f**), plus a summary of quantification (**g**). Data are representative of 2 similar experiments. **h** NE levels measured in ex vivo ATM culture medium (left panel) and cell lysates (right panel); SVF from 5 mice in each group were pooled and sorted to obtain CD45 + CD11b + F4/80+ cells, and NE was measured in 2 or 3 technical replicates). Data are presented as mean ± s.e.m. Statistical significance was assessed by unpaired two-sided *t*-test (**a**–**c** and **g**) and two-way ANOVA using Tukey’s multiple comparisons test (**h**). **i** Linear regression analysis of AIF1 and MAOA or ALDH1L2 expression in subcutaneous adipose tissues of clinical participants with excess weight or obesity (*n* = 11), with significance assessed by Pearson’s correlation. Curved lines indicate 95% confidence intervals. *n* represents a number of biologically independent animal or human participants unless otherwise noted. Source data are provided as a Source Data file.

Macrophages exhibit both organ- and niche-specific gene expression and function^5,39^ so we asked if these AIF1-mediated effects on MAOA expression in BMMs extended to macrophages in adipose tissues. Compared to controls, RNA extracted from intact tissues had mildly lower *Maoa* expression levels in BAT and iWAT of *Aif1*^*−/−*^ mice fed with either CD or HFD, while reduced *Aldh1/2* was seen only in BAT and iWAT of HFD-fed mice (Supplementary Fig. 8b); tissue fractionation indicated that this decrease in *Maoa* expression occurred in the stromal-vascular fraction (SVF)—which would include macrophages—and not in adipocytes (Supplementary Fig. 8c). This difference was particularly apparent in the iWAT SVF, which showed a multi-fold increase in *Maoa* expression with HFD in WT mice that was effectively blunted by AIF1 deficiency. Consistent with the decrease in *Maoa* gene expression in *Aif1*^*−/−*^ BMMs (Fig. 4d, e), we found a reduction in *Maoa* transcript levels in sorted CD45^+^;CD11b^+^;F4/80^+^ ATM populations collected from BAT and iWAT, but no significant change in eWAT (Fig. 5a–c; gating strategy is shown in Supplementary Fig. 9a–d). Furthermore, levels of MAOA^+^ ATMs in the BAT and iWAT, but not eWAT, of HFD-fed *Aif1*^*−/−*^ mice were approximately 50% lower than corresponding WT controls (Fig. 5d–g). We also observed that *Aif1*^*−/−*^ ATMs from BAT and iWAT (Fig. 5h) contained more NE, suggesting that loss of AIF1 impairs the ability of these cells to clear the catecholamine. This differential catabolism was not observed in AIF1-deficient ATMs sorted from eWAT (Fig. 5h). Likewise, adipose tissue monocytes and dendritic cell populations from AIF1-deficient mice showed no differences in the level of MAOA^+^ cells compared to controls (Supplementary Fig. 10a–h).

AIF1, known as Iba1 (ionized calcium binding adaptor molecule-1)^40^ in the neuroscience literature, is also expressed in microglia. As noted previously, we did not find significantly higher levels of NE or 5-HT in the brains of mice lacking AIF1. Consistent with this observation, overall brain levels of MAOA were not changed by the loss of AIF1, perhaps because *Maoa* expression in microglia is already notably low among brain cells in the mouse (https://www.brainrnaseq.org)^41,42^ (Supplementary Fig. 11). As noted previously, such organ-dependent heterogeneity of gene expression among cells of monocytic lineage is well known^5^.

Adipose tissue-resident macrophages have been linked to non-classical homeostatic functions such as control of energy expenditure, whereas the recruited proinflammatory macrophages in obese adipose tissue promote inflammation and insulin resistance^6^. We looked for effects of AIF1 on macrophage polarization in adipose tissue and obesity-associated insulin resistance, and found no significant differences in M1 vs. M2 marker expression in BAT from WT and *Aif1*^*−/−*^ mice fed CD or HFD; circulating adipokines including adiponectin and resistin were also not different (Supplementary Fig. 12a, b, e). In eWAT, however, we noted increases in M2 and decreases in M1 marker expression of *Aif1*^*−/−*^ mice compared to controls on both diets (Supplementary 12c, d); levels of serum lL-6 on HFD and IL-12p70 on both diets were significantly lower (Supplementary Fig. 12f, g). These observations suggest that on either diet, AIF1 deficiency promotes an M2-like phenotype of eWAT macrophages plus effects on circulating cytokines that may contribute to improved glucose tolerance and insulin sensitivity (Fig. 1). Finally, to assess the potential relevance of this AIF1–MAOA axis to human obesity, we evaluated the expression of AIF1 and NE degradation pathway genes in discarded subcutaneous adipose tissues from participants with weight excess or obesity (BMI range 28.5–34.3, Supplementary Table 2). These studies showed a direct correlation of adipose tissue *AIF1* transcript levels with those of NE degradation pathway genes, including *MAOA* and *ALDH1L2* (Fig. 5i).

## Discussion

Control of sympathetic signaling activity in adipose tissues is emerging as a key macrophage function affecting metabolism. Recent studies describe the close physical association of macrophages with nerve fibers in BAT^9^, visceral adipose tissue (VAT)^10^, and iWAT^11^. Deletion of *Mecp2* in CX3CR1^+^ macrophages impaired BAT innervation during development, leading in turn to low NE levels, insufficient thermogenic activity, and obesity^9^. Aging-associated increases in macrophage inflammasome activation in VAT promoted the expression of catecholamine catabolism genes, including *Maoa* and *Comt*, which in turn decreased NE levels and lipolysis in older mice^10^. Sympathetic-associated macrophages (SAMs) characterized in iWAT have been shown to take up NE selectively via the SLC6A2 transporter and degrade it via MAOA; transplantation of irradiated *ob/ob* mice with bone marrow cells lacking SLC6A2 resulted in higher circulating NE, together with evidence for higher BAT activities and improved ability to lose weight^11^.

Our studies have identified a striking resistance to obesity accompanied by improved glucose handling in mice deficient in AIF1, a protein preferentially expressed in cells of the myeloid lineage. Notable aspects of this phenotype include increased energy expenditure, sustained lean mass and food intake, and the lack of a consistent and corresponding increase in physical activity. Our results indicate that AIF1 is required for the expansion of brown and white adipose tissue depots in mice eating an HFD, and suggest that it mediates this function by suppressing thermogenic programs in these tissues: mice lacking AIF1 show increased thermogenic gene expression and β-AR signaling in both BAT and WAT, accompanied by higher levels of NE that likely result from the lower expression of genes required for catecholamine catabolism, including *Maoa*, by macrophages in adipose tissues. The potential relevance of AIF1-dependent catecholamine catabolism to human obesity is supported by the positive correlation of *AIF1* with *MAOA* and *ALDH1L2* transcript levels in adipose tissues from participants with weight excess or obesity.

Interestingly, mouse core body temperatures obtained during indirect calorimetry studies did not differ according to genotype at 22 or 30 °C but were mildly lower in *Aif1*^*−/−*^ mice housed at 10 °C (Fig. 2e, g, i) on either CD or HFD. This last finding suggests that loss of AIF1 expression impairs the animals’ ability to defend body temperature in response to extended cold stress, despite higher energy expenditure. Understanding why AIF1-deficient mice fail to maintain body temperature under these conditions will require further investigation; possibilities include increased energy loss (e.g., dysfunctional vasoregulation, which could in turn reflect altered CNS or local vasomotor controls^43^), defects in alternative (non-UCP1-dependent) mechanisms of thermogenesis, or perhaps inadequate replenishment of intracellular lipid stores needed to meet the increased demands of heightened brown/beige adipocyte metabolism during the course of the 5-day experiment^44^.

We also note that these studies rely on mice with germline *Aif1* inactivation, which constrains some mechanistic interpretations. The identified differences in BAT and iWAT macrophage function and increased local levels of NE accompanying AIF1 loss—or even potential changes in adipocyte metabolic capability—are consistent with effects within these adipose depots. Although AIF1 is preferentially expressed in the myeloid lineage and our cell culture and bone marrow transplantation findings primarily implicate macrophages, we cannot fully exclude intrinsic effects of AIF1 deficiency on other cell types within these depots—e.g., adipocytes, vascular cells, or nerves—that might contribute to the phenotype. In addition, AIF1—or Iba1^40^ in the neuroscience literature—is commonly used as a marker of microglia, as noted previously. Conceivably, increased sympathetic nervous system activity originating in the CNS could contribute to higher adipose tissue NE levels seen in *Aif1*^*−/−*^ mice—though such a mechanism would not readily explain the observed decreases in MAOA + macrophages and associated NE catabolic capacity. In the present context, we did not find increases in NE or 5-HT levels in WT and *Aif1*^*−/−*^ brain samples (Fig. 3b, Supplementary Fig. 6b) that might explain obesity resistance, nor in circulating factors that would accompany activation of the hypothalamic–pituitary–adrenal axis (Fig. 3). The nature of AIF1/Iba1 contributions to microglial and CNS function remain largely to be elucidated^16,45^, and our results do not exclude the possibility that AIF1 might affect neurotransmitter levels in a localized and discrete way within the brain that could impinge on metabolism. Noting again that *Maoa* transcript levels in mouse microglia are well below those in other brain cell types, we surmise that the effects of AIF1/Iba1 on brain chemistry, if any, are likely to differ mechanistically from those we have identified in ATMs. In turn, how such effects might contribute to the control of systemic metabolism remains a point for speculation at present. It is also important to note that the mouse model studies reported here are limited to male mice, and the findings are not necessarily generalizable to female mice.

Finally, AIF1 has been characterized as an actin-bundling cytoplasmic protein, and loss of function studies indicate that macrophages lacking AIF1 have impaired cell migration and phagocytosis^46–48^. The list of validated protein interactions with AIF1 is quite limited but includes actin and l-plastin^49–51^. While a multitude of cytoplasmic signaling mechanisms, including actin polymerization^52^, converge directly or indirectly on the cell nucleus to exert effects on gene expression, there is little specific information about actin-bundling proteins in this context^53^, and the nature of the positive correlation of AIF1 with MAOA expression remains to be determined. Nevertheless, the links between AIF1 and clinical adiposity, the relatively restricted pattern of AIF1 expression, and the apparently benign baseline phenotype of *Aif1*^*−/−*^ mice suggest that methods to interrupt its effects on gene expression could provide a useful approach toward the mitigation of obesity.

## Methods

This research complies with all relevant ethical regulations. Animal experiments were conducted in accordance with NIH guidelines under protocols approved by the Institutional Animal Care and Use Committee of the Albert Einstein College of Medicine. Collection and analysis of clinically derived but discarded and deidentified samples were conducted following established guidelines of the New York University (NYU) Institutional Review Board (IRB Registration No. 00000310) and the NYU Human Research Protections Program. This policy permitted PI self-certification and waiver of consent for this use of discarded, deidentified materials, which qualified as non-human subjects research. Figures were prepared using Illustrator software version 23 (Adobe).

### Mice

Mice were housed in groups of up to 5 in plastic isolator cages within the specific pathogen-free Barrier Facility of the Albert Einstein College of Medicine. AIF1-deficient mice^14^ and littermate controls were backcrossed for 14 generations to the C57BL/6J strain, maintained in standard 12 h light-dark cycles (6 AM–6 PM) at 22 °C, humidity range 30–70%, and fed with irradiated control chow diet (21.6% kcals from fat, mouse diet 20-5058, Pico Lab) or an HFD (60% kcals from fat, D12492i, Research Diets). The age of mice used in these experiments ranged between 6 and 24 weeks. All animal experiments were conducted in accordance with NIH guidelines under protocols approved by the Institutional Animal Care and Use Committee of the Albert Einstein College of Medicine. Humane endpoints for removal from the study were established, and animal welfare was monitored on a daily basis for such signs of disease or discomfort, including lethargy, focal neurologic signs, unhealing skin lesions, or significant weight loss. Animals showing persistence of such signs for 48 h without improvement were removed from the study and euthanized. All experiments reported here were performed with male mice.

### Metabolic phenotyping

Mouse body weights were measured starting at age 8 weeks and every 2 weeks thereafter for the next 16 weeks. Whole body fat and lean masses were assessed by MRI (whole body composition analysis, Echo-MRI). Whole body scanning, eWAT, and iWAT adipose tissue masses were evaluated by computerized tomography scanning (La Theta CT, Hitachi Aloka). Tissue mass was assessed by weighing wet tissues at the endpoint of experiments. Metabolic activities (O_2_ consumption, CO_2_ release, energy expenditure, physical activity, food intake, and core body temperature) were measured in metabolic chambers (Columbus Instruments) under cold (10 °C), ambient (22 °C), or thermoneutral (30 °C) conditions. For glucose tolerance tests, animals were fasted overnight prior to the administration of intraperitoneal glucose (1.5 g/kg of lean mass, Sigma); systemic glucose levels were measured before and after glucose administration at the timepoints indicated. Insulin resistance was measured by hyperinsulinemic-euglycemic clamp studies, as per published protocols^54^.

### Immunoblot analyses

Immunoblot analysis was performed to detect AIF1, phosphorylated and total HSL, and MAOA. In brief, tissues or cells were homogenized in RIPA buffer containing cOmplete^TM^ protease and phosphatase inhibitors (both from Roche). Soluble protein in the lysates was quantified and samples were subjected to gel electrophoresis. For the evaluation of AIF1 and respiratory complex proteins, we used 10–20% tricine gels (Novex) and transferred proteins onto 0.2 μm PVDF membrane. For other target proteins, we used 4–12% Bis–Tris gels and 0.4 μm PVDF membrane. Membranes were blocked with Odyssey blocking buffer (LiCOR), and incubated with primary antibodies overnight at 4 °C, followed by secondary antibodies for 1 h at RT. Primary antibodies used were rabbit anti-AIF1 (#016-20001, 1:500 dilution, Wako), rabbit anti-AIF1 (#ab178847, 1:500 dilution, Abcam), rabbit anti-Phospho-HSL (#4126, 1:500 dilution, Cell Signaling), rabbit anti-HSL (1:500 dilution, Cell Signaling), rabbit anti-MAOA (#ab126751, 1:2000 dilution, Abcam), mouse anti-OXPHOS proteins (#ab110413, 1:150 dilution, Abcam), and mouse anti-β-actin (1:5000 dilution, Abcam). For immunoblot analyses other than MAOA, anti-rabbit IRDye@800 CW (1:5000 dilution, Li-COR) and anti-mouse-IRDye@680 CW (1:5000 dilution, Li-COR) were used as secondary antibodies, and signals were detected using an Odyssey scanner. MAOA detection was performed using goat-anti-rabbit IgG-horseradish peroxidase (1:2000 dilution, Jackson ImmunoResearch) and ECL Western Blotting Substrate (Pierce). Additional antibody information is provided in Supplementary Table 3.

### Quantitative RT-PCR

Total mRNA was isolated from tissues and cells using Trizol (Invitrogen), and cDNA was prepared using Super Script III reverse transcriptase (Invitrogen). Gene expression levels were quantified by real-time PCR using an SYBR green PCR master mix and a Roche Light Cycler system. Gene expression on sorted ATMs was assessed using the Cells-to-CT^TM^ 1-step Power SYBR Green kit (Ambion Life Technologies) and a ViiA 7 Applied Biosystems cycler system. Quantification of the PCR signals of each sample was performed by evaluating the relative expression of each gene by normalizing it to the reference gene β*-actin* using the ΔΔCt method. Specific cDNA primer sequences used are provided in Supplementary Table 4.

### Histology and immunofluorescence

Tissues were isolated at the indicated time points and fixed in 4% paraformaldehyde. Paraffin sections (0.5 µm) were subjected to hematoxylin–eosin staining for histologic analysis. UCP1 expression was assessed in antigen-retrieved paraffin sections by blocking with 5% normal goat serum and incubating overnight with rabbit anti-UCP1 (1:500, ThermoFisher), followed by anti-rabbit IgG 594 (Life Technologies) for 1 h at RT. Images were captured using a Zeiss Axio imager and analyzed by ImageJ software. Antibody information is provided in Supplementary Table 3.

### Flow cytometry

Adipose tissues from brown, iWAT, and eWAT depots were harvested and digested with Collagenase D (Roche) for 1 h at 37 °C with shaking. Cell suspensions were filtered through a 70 μm filter and centrifuged at 450×*g* for 5 min. Single-cell suspensions were lysed to remove red blood cells and stained for Zombie Yellow (1:100 dilution, BioLegend) to label the dead cells. Cells were stained with anti-mouse CD16/CD32 (1:100 dilution, BD Biosciences) antibody to Fc-block followed by CD45-APC-F750, CD11b-PE (Biolegend), F4/80-A647 (Biorad), CD11c-BUV395 (BD Biosciences), anti-rabbit AIF1-unlabeled (1:100 dilution, Abcam) and anti-rabbit MAOA-unlabeled (1:100 dilution, Abcam). For intracellular staining of AIF1 or MAOA, anti-rabbit 594 (1:300 dilution, Invitrogen) was used as a secondary antibody. Stained cells were acquired on an LSR II cytometer (BD Biosciences) using BD FACS Diva™ software and the data were analyzed using FlowJo software (FlowJo, LLC). ATMs were sorted using an Aria cell sorter (BD Biosciences). Additional antibody information is provided in Supplementary Table 3.

### Bone marrow-derived macrophage generation and culture

Bone marrow from the femur and tibia of 8-week-old WT and *Aif1*^*−/−*^ mice were isolated and cells were differentiated into macrophages^55^ and immortalized with SV40 large T-antigen using a standard protocol^56^. BMMs were maintained in α-MEM containing 10% FBS, 120 ng/mL CSF-1 (Chiron Corp.), penicillin–streptomycin, and l-glutamine (Gibco).

### Bone marrow transplantation

Total bone marrow cells were extracted from femurs and tibiae of 6–8-week-old WT (CD45.1 leukocyte marker, Jax stock #002014) or *Aif1*^−/−^ (CD45.2 leukocyte marker) male donor mice, and red blood cells were removed by incubation with RBC Lysis Buffer (Biolegend) for 5 min at 4 °C prior to injection. Recipient WT and *Aif1*^−/−^ male mice aged 4–6 weeks were irradiated with 1000 rad split into two doses with a 4-h interval (Mark I-68A 137Cs irradiator, JL Shepherd), and 1 × 10^7^ bone marrow cells were injected intravenously (WT cells to *Aif1*^−/−^ recipients and *Aif1*^−/−^ cells to WT recipients). After 6 weeks of recovery, transplanted mice were assigned to either CD or HFD; body weight was measured every 2 weeks thereafter, and fat mass and lean mass were assessed by MRI at the end of 6 weeks on diet. Mice were monitored for poor recovery or limited engraftment, with flow cytometry used to assess transplantation efficiency according to CD45.1 vs. CD45.2 representation in peripheral blood. Only mice with >75% transplantation efficiency were included in the analysis.

### RNA isolation, library construction, and analysis

Total RNA was isolated using TRIzol Reagent (Invitrogen), and the integrity was validated using an Agilent 2100 Bioanalyzer. Total RNA (200 ng) was used to prepare libraries using KAPA stranded RNA-seq kit with RiboErase as described (Kapa Biosystems, #KR1151-v3.15). Briefly, total RNA was subjected to rRNA depletion and double-stranded cDNA (dscDNA) synthesis followed by fragmentation. A-tailing was done at the 3’ end of the dscDNA library fragments and then subjected to library amplification followed by adapter ligation for Illumina sequencing. Control RNA was spiked in (ERCC RNA Spike-In control mixes, Life Technologies) as an internal control for the library preparation and sequencing. PCR-amplified cDNA libraries were quantified on the Agilent 2100 Bioanalyzer and diluted to 10 pM for cluster generation and sequencing. Single-end sequencing was performed on Illumina’s Platform NEXTSeq500 using NSQ® 500 hi-Output KT v2 (75 CYS- (Cat # FC-404-2005)). The quality of the sequence reads was assessed using FastQC. FastQC files were trimmed of their adapters with trim_galore (v0.5.0), fastqc (v0.11.5), and cutadapt (v1.15) under default parameters. Reads were aligned to the GRCm38 mouse genome (https://www.ncbi.nlm.nih.gov/assembly/GCF_000001635.20/) with Tophat (v2.0.13)^57^ with parameters allowing a read to be mapped to at most one location: “–no-coverage-search -p 1 -g1”. Gene hits were counted with HTseq (v0.6.1)^58^ under default parameters using release 84 of the Mus_musculus GRCm38 GTF annotation file. Differential expression analysis was performed in R/Bioconductor following the DESeq2 workflow as described^59^ and annotated with biomaRt^60^. Pathway analysis was performed using Ingenuity version 01-14 (Qiagen).

### NE measurement

Adipose tissues, liver, brain, heart, and serum were collected at specific timepoints. NE and 5-HT levels were measured using a standard protocol^61^. Tissue pieces were homogenized using a tissue dismembrator in buffer (0.1 M trichloroacetic acid, 10^−2^ M sodium acetate, 10^−4^ M EDTA and 10.5% methanol (pH 3.8)), spun in a microcentrifuge at 10,000×*g* for 20 min at 4 °C, and the supernatant was removed for biogenic monoamine analysis using specific liquid chromatography/mass spectrometry (LC/MS) following derivatization of analytes with benzoyl chloride (BZC). Tissue or cell extract (20 µL) was mixed with 60 µL of 80% acetonitrile in water and 0.5% formic acid, vortexed for 20 s, and spun at 10,000×*g* for 2 min at 20 °C. The supernatant (5 µL) was combined in an LC/MS vial with 10 µL each of 500 mM NaCO_3_ (aq) and 2% BZC in acetonitrile. The reaction was stopped after 2 min by adding 20 µL internal standard solution (20% acetonitrile containing 3% sulfuric acid) and 40 µL H_2_O. The samples were then subjected to LC/MS analysis. LC was performed on a 2.0 × 100 mm, 1.7 µm particle Kinetix biphenyl column (Phenomenex, Torrance, CA, USA) using a Waters Acquity UPLC (Waters Corporation, Milford, MA, USA). Mobile phase A was 0.15% aqueous formic acid and mobile phase B was acetonitrile. Samples were separated by a gradient of 98–5% of mobile phase A over 6 min at a flow rate of 450 µL/min prior to delivery to a SCIEX 6500 and QTrap mass spectrometer.

### Respirometry

Tissue bioenergetics were assessed using a Seahorse respirometer (Agilent)^62^. Briefly, BAT and iWAT of 8–12 weeks old animals treated with vehicle or propranolol (1 mg/kg) for 3 consecutive days were collected and rinsed with Krebs–Henseleit buffer (KHB) (111 mM NaCl, 4.7 mM KCl, 2 mM MgSO_4_, 1.2 mM Na_2_HPO_4_, 0.5 mM carnitine, 2.5 mM glucose and 10 mM sodium pyruvate). Respective tissue slices (6–10 mg) were transferred to individual wells of an XF24 plate, stabilized by islet capture screens (Seahorse Bioscience), and KHB (450 µL) was added to each well. Digitonin was added to enhance plasma membrane permeability. Basal oxygen consumption rates (OCR) were determined at 37 °C according to the following protocol: basal readings were recorded every 2 min for 10 readings, followed by exposure to digitonin. Subsequent readings were recorded after 2 min mixing and 2 min rest. Basal OCR values were normalized to individual tissue weights.

To evaluate oxygen consumption of in vitro-differentiated brown and beige adipocytes, SVFs were obtained from BAT of 5–6-week-old WT and *Aif1*^−/−^ mice (*n* = 6 each) and from iWAT of 15-week-old WT and *Aif1*^−/−^ mice (*n* = 3 each). In brief, adipose tissues were dissociated with collagenase D (1 mg/mL) and preadipocytes were plated in six-well plates and grown for 3 days in DMEM containing 10% FBS. After this period, preadipocytes were plated in a 96-well Seahorse plate (10,000 cells/well) and differentiation was induced with DMEM-F12 media containing 10% FBS, troglitazone (5 μM), 3-isobutyl-1-methylxanthine (IBMX, 0.5 mM), dexamethasone (1 µM), insulin (5 µg/mL), and triiodothyronine (T3, 50 nM). After 2 days, media were changed to a maintenance formulation with troglitazone (5 μM), insulin (5 µg/mL), and T3 (50 nM). After 6 days, oxygen consumption was assessed with the Seahorse respirometer, with the administration of oligomycin (2 µM) to distinguish total and ATP-linked activity. Total protein was isolated from each well and quantified for normalization after the assay.

### Enzymatic activity measurement and NE clearance in BMMs and ATMs

MAOA activity in cultured WT and *Aif1*^*−/−*^ BMMs was assessed using an assay kit (K795-100, BioVision). Briefly, total cellular lysates (250 µg) were assessed in a 96-well plate in the presence or absence of the MAOA inhibitor clorgyline (10 µM). The activity was measured by a fluorescence plate reader (Varioskan LUX multimode Reader, Thermo Fisher) using wavelength settings of 535/587 nm (excitement/emission), and data were presented as relative fluorescence units (RFU). To assess NE clearance, BMMs (0.25 × 10^6^/well) in 48-well plates were exposed to exogenous NE (2 µM) ± clorgyline (10 µM). After 2 h, the medium was removed, and the cells were washed with PBS and homogenized in 0.01 N HCl in the presence of 1 mM EDTA and 4 mM sodium metabisulphite and stored at −80 °C. NE concentrations in the lysates were assessed by LC/MS.

NE clearance of ATMs was assessed by plating 600–1000 cells of FACS-sorted ATMs on 96-well plates in the presence or absence of clorgyline (10 µM). After 2–3 h, the medium was removed, and cells were washed with PBS and homogenized in 0.01 N HCl in the presence of 1 mM EDTA and 4 mM sodium metabisulphite and stored at −80 °C. NE concentrations in the lysates were assessed by LC/MS.

### Serum analysis

Adipokines such as resistin and adiponectin, Leptin, and corticosterone were measured using ELISA Kit (R&D). Serum cytokine levels were evaluated by magnetic Luminex assay (Sigma-Millipore).

### Human adipose tissue analysis

Discarded, de-identified human adipose tissues were obtained from clinical procedures, as described in Supplementary Table 2. Tissue samples were snap-frozen and stored at −80 °C until homogenization in Trizol (Invitrogen) for RNA isolation and quantitative RT-PCR analysis.

### Statistical analysis

Statistical analyses were performed using Prism 7 software (GraphPad). Data are represented as mean ± s.e.m., with significance assessed by unpaired two-sided Student’s *t*-test for single comparisons and one-way or two-way ANOVA followed by Tukey or Sidak multiple comparison tests. For analysis of gene expression in human samples, the correlation coefficient (*r*) was obtained using Pearson’s method. Values were considered statistically significant for *P* ≤ 0.05.

### Reporting summary

Further information on research design is available in the Nature Portfolio Reporting Summary linked to this article.

## Supplementary information

Supplementary information

Description of Additional Supplementary Files

Supplementary Data 1

Reporting Summary

## Data Availability

The RNA-seq data for this study is available from the Gene Expression Omnibus (GEO; GSE133278. The metabolic and other gene expression data generated in this study are provided in the Supplementary Information or Source Data files, both provided with this paper. The raw data from human participants (beyond what is shown) are protected and not available due to data privacy considerations. Source data are provided with this paper.

## Source data

Source Data
