# Supplementary material for: Increased adipose catecholamine levels and protection from obesity with loss of Allograft Inflammatory Factor-1
Source: Nat Commun. 2023 Jan 3;14:38. doi: 10.1038/s41467-022-35683-7 (PMC9810600; doi:10.1038/s41467-022-35683-7)
Supplement: Supplementary file 1 — Supplementary information [file 41467_2022_35683_MOESM1_ESM.pdf]

Supplementary Figures

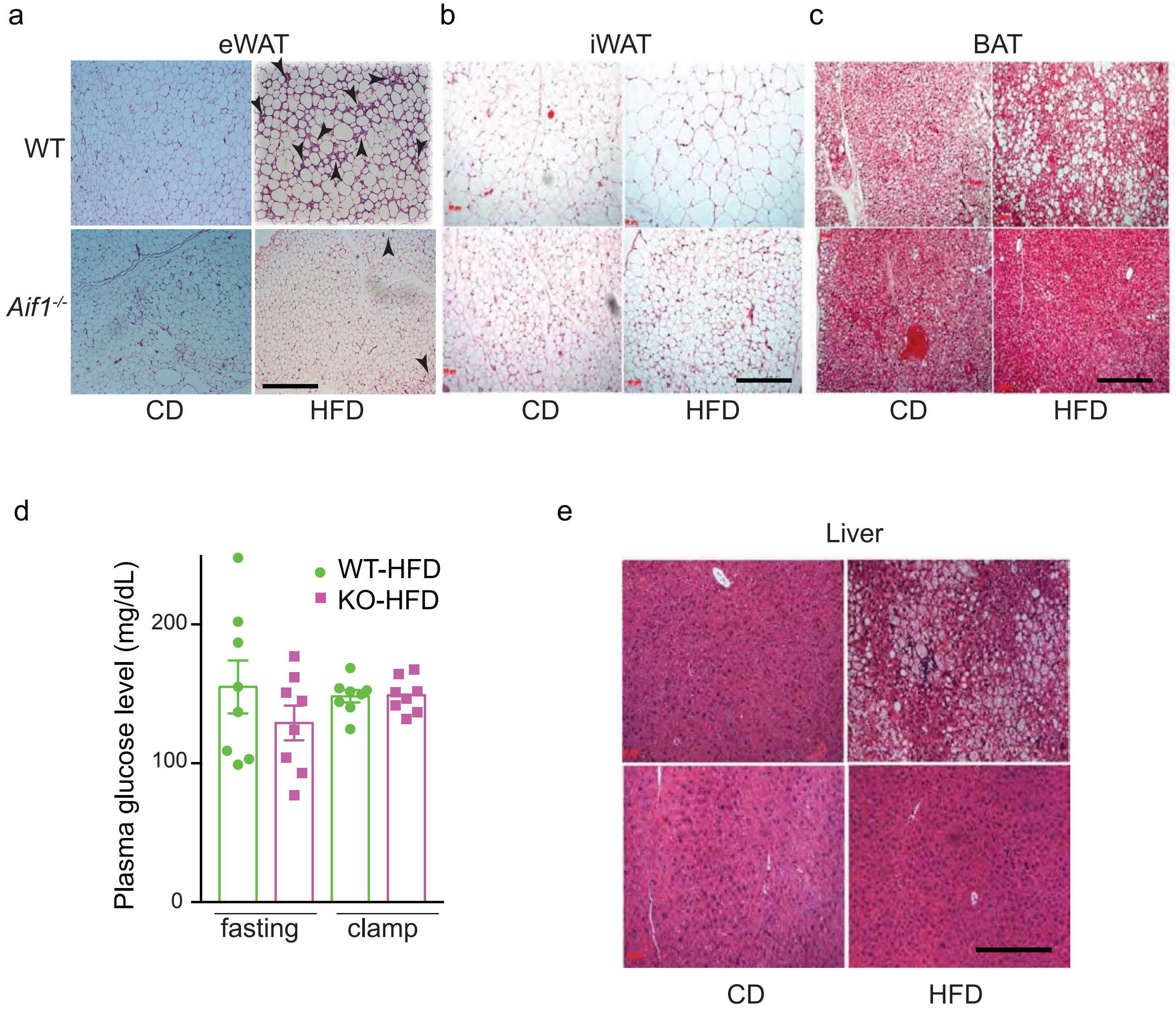

Supplementary Fig. 1. Effect of AIF1 loss on adipose tissues and liver. Starting at 8 weeks of age, WT and *Aif1*$^{-/-}$ (KO) mice were fed with CD or HFD for 16 weeks prior to evaluation. **a**–**c**, Hematoxylin-eosin staining of eWAT (**a**), iWAT (**b**), and BAT (**c**). Black arrows indicate the crown-like structures between the adipocytes. **d** Plasma glucose levels with fasting and during clamp experiment. n=8 biologically independent animals per group; presented as mean ± s.e.m. **e** Hematoxylin-eosin stained sections of liver. Images are representative of n=5 animals per group. Scale bar, 10 μm. Data are representative of three similar experiments. Source data are provided as a Source Data file.

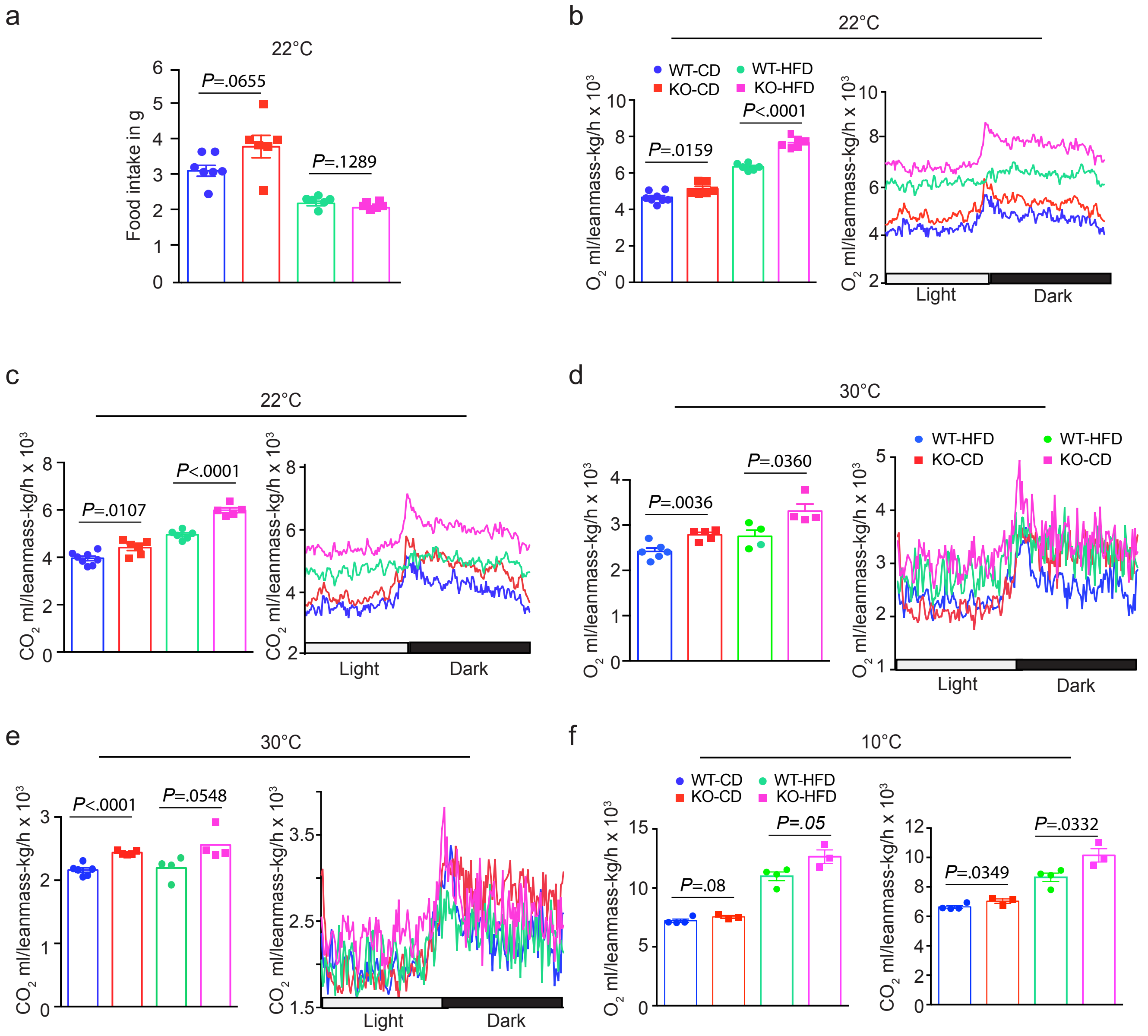

Supplementary Fig. 2. AIF1 deficiency in mice increases O_2 consumption and CO_2 release. Metabolic characteristics of AIF1-deficient and control mice (n=4–6 per group) fed with CD or HFD for 16 weeks were measured using metabolic cages at ambient temperature (22°C), thermoneutrality (30°C), or cold (10°C). **a** Food intake, showing mean values collected over 4 days at 22°C (n: WT-CD, 7; KO-CD, 6; WT-HFD, 6; KO-HFD, 6). **b** O_2 consumption (n: WT-CD, 8; KO-CD, 6; WT-HFD, 6; KO-HFD, 6). and **c** CO_2 release (n: WT-CD, 8; KO-CD, 6; WT-HFD, 6; KO-HFD, 5).measured at 22°C, showing mean values collected over 4 days (left panel) and over a 24h light-dark cycle (right panel). Data are representative of three similar experiments. **d**, **e** Similar assessments, but with mice housed at 30°C (n: WT-CD, 6; KO-CD, 5; WT-HFD, 4; KO-HFD, 4) **f** O_2 consumption and CO_2 release measured at 10°C, showing mean values collected over 5 days (n: WT-CD, 4; KO-CD, 3; WT-HFD, 4; KO-HFD, 3). Data are presented as mean ± s.e.m.; significance was evaluated by two-sided unpaired Student's t-test. n represents number of biologically independent animals. Source data are provided as a Source Data file.

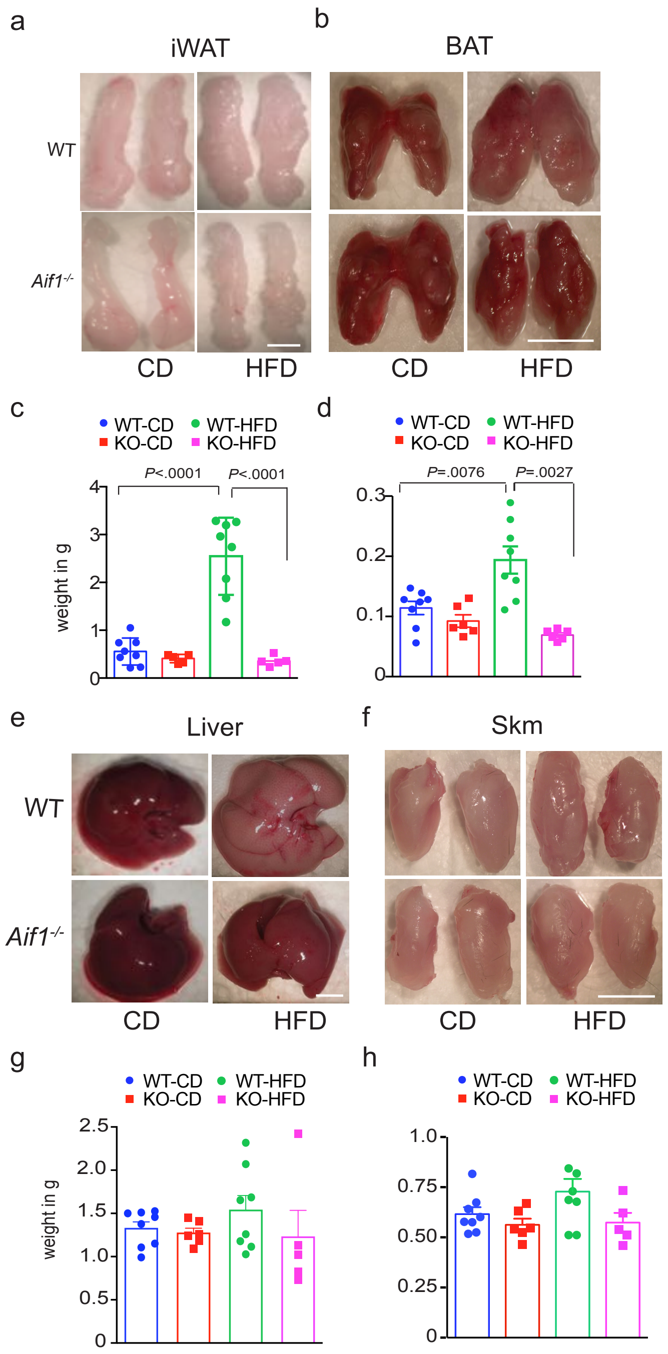

Supplementary Fig. 3. AIF1 deficiency suppresses HFD-mediated increases in adipose tissue mass, without changes in liver or skeletal muscle mass. WT and *Aif1*$^{-/-}$ mice were fed with CD or HFD for 16 weeks prior to evaluation. **a**–**d** Macroscopic images of iWAT (**a**) and BAT (**b**) with respective quantification in **c** (n: WT-CD, 8, KO-CD, 5; WT-HFD, 8, KO-HFD, 5) and **d** (n: WT-CD, 8, KO-CD, 6; WT-HFD, 8, KO-HFD, 6). **e**–**h** Macroscopic images of liver (**e**), and skeletal muscle (skm) (**f**), with respective quantification in **g**, **h**.(n: WT-CD, 8, KO-CD, 6; WT-HFD, 8, KO-HFD, 5). Images are representative of n=5 per group. Data are presented as mean ± s.e.m and representative of 3 similar experiments. Scale bars = 5 mm. Significance assessed by one-way ANOVA with Tukey's multiple comparisons test. n represents the number of biologically independent animals. Source data are provided as a Source Data file.

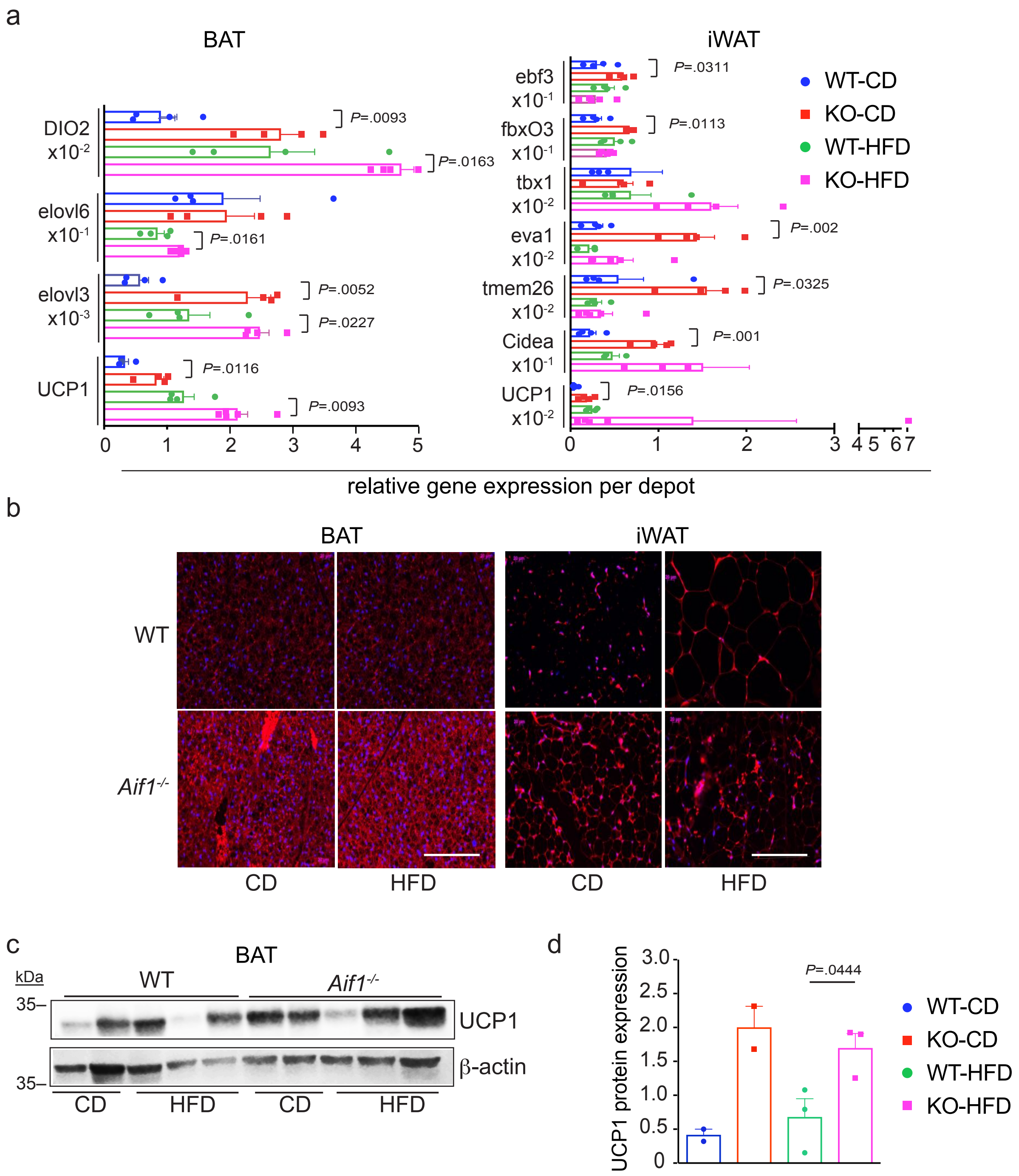

Supplementary Fig. 4. Adipose tissues lacking AIF1 have higher expression of thermogenic genes and UCP1 protein. WT and *Aif1*$^{-/-}$ mice (age 8 weeks, n=3–5 per group) were fed with CD or HFD for 16 weeks. Total RNA was isolated from adipose tissues for analysis of gene expression. **a** Thermogenic gene expression in BAT (left) and iWAT (right) normalized to total tissue weight on CD and HFD and represented as relative expression per depot.depot (n: WT-CD, 4, KO-CD, 4; WT-HFD, 5, KO-HFD, 5). **b**–**d** UCP1 protein expression assessed by immunofluorescence in BAT (**b**, left) and iWAT (**b**, right); scale bars,10 μm) and by immunoblot in BAT (**c**), with quantification (**d)** (n: WT-CD, 2, KO-CD, 2; WT-HFD, 3, KO-HFD, 3). Images are representative of n=5 per group. Quantitative data are presented as mean ± s.e.m, and are representative of two similar experiments. Significance assessed by unpaired two-sided Student's t-test (a,d). n represents number of biologically independent animals. Source data are provided as a Source Data file.

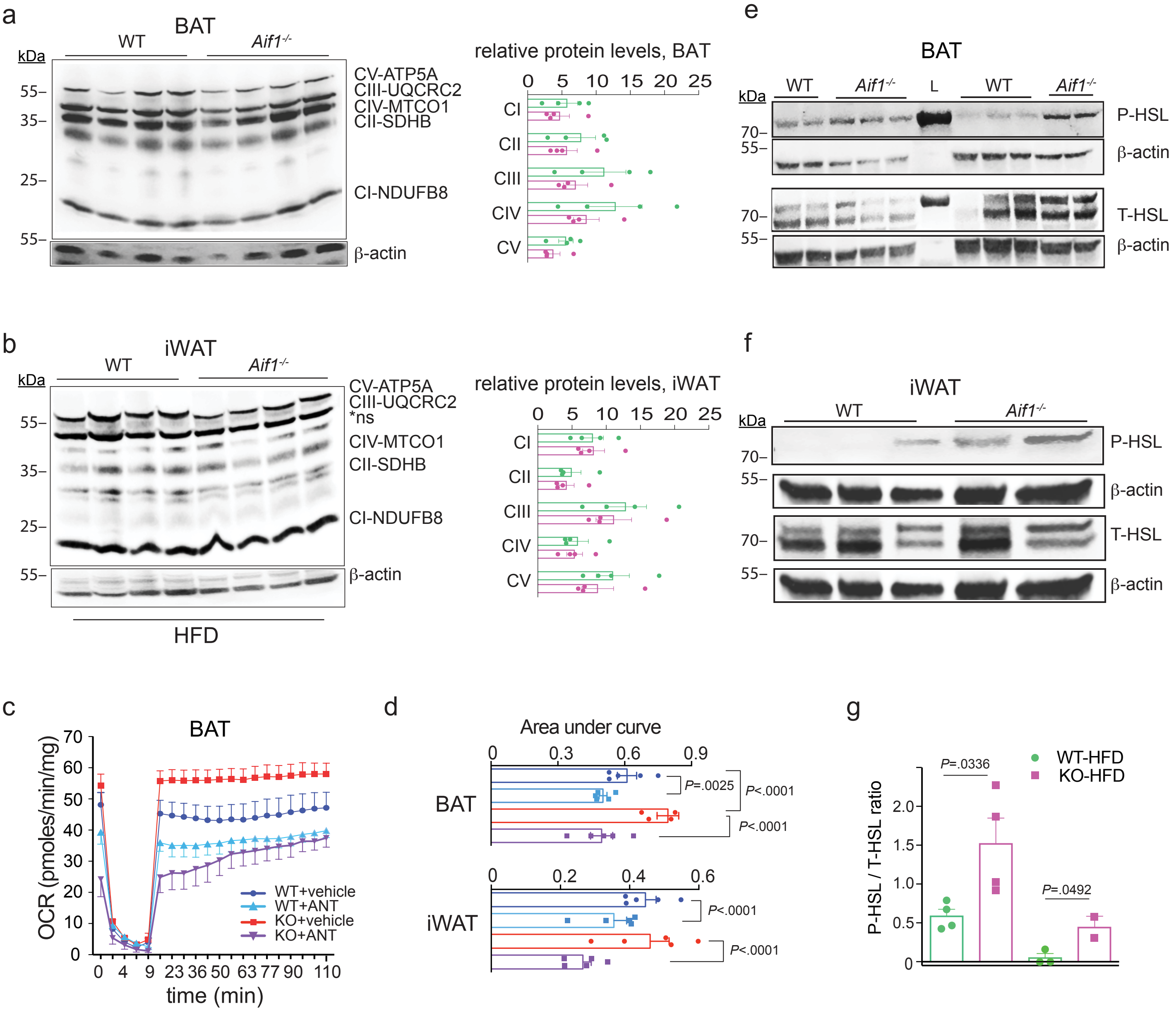

Supplementary Fig. 5. AIF1 deficiency in mice increases adipose tissue oxygen consumption and β-adrenergic signaling without affecting mitochondrial content. **a,b** OXPHOS protein levels in BAT (**a**) and iWAT (**b**) on HFD by immunoblot (left); levels were not signficantly different (right panels), n=4 independent animals per group. *ns – non specific band. **c,d** Oxygen consumption rate (OCR) of BAT measured using a Seahorse extracellular flux assay. Mice aged 6–8 weeks were treated with vehicle or propranolol (antagonist (ANT), 1 mg/kg) prior to tissue harvest (n=5 independent animals per group). **d**, area under curve (AUC) measured from OCR of BAT or iWAT, as indicated; groups as in panel **c. e–g**, Immunoblot analysis of phospho-hormone sensitive lipase (P-HSL) and total-HSL (T-HSL) levels in **e**, BAT, and **f**, iWAT, and quantitative analysis in (**g**). BAT, n=4 and iWAT, n=2 WT and 3 KO. Data are presented as mean ± s.e.m. Significance assessed by unpaired two-sided Student's t-test (**a,b,g**) or one way ANOVA with Tukey's multiple comparisons test, **d.** Data are representative of two similar experi-ments, with n indicating the number of biologically independent animals. Source data are provided as a Source Data file.

.

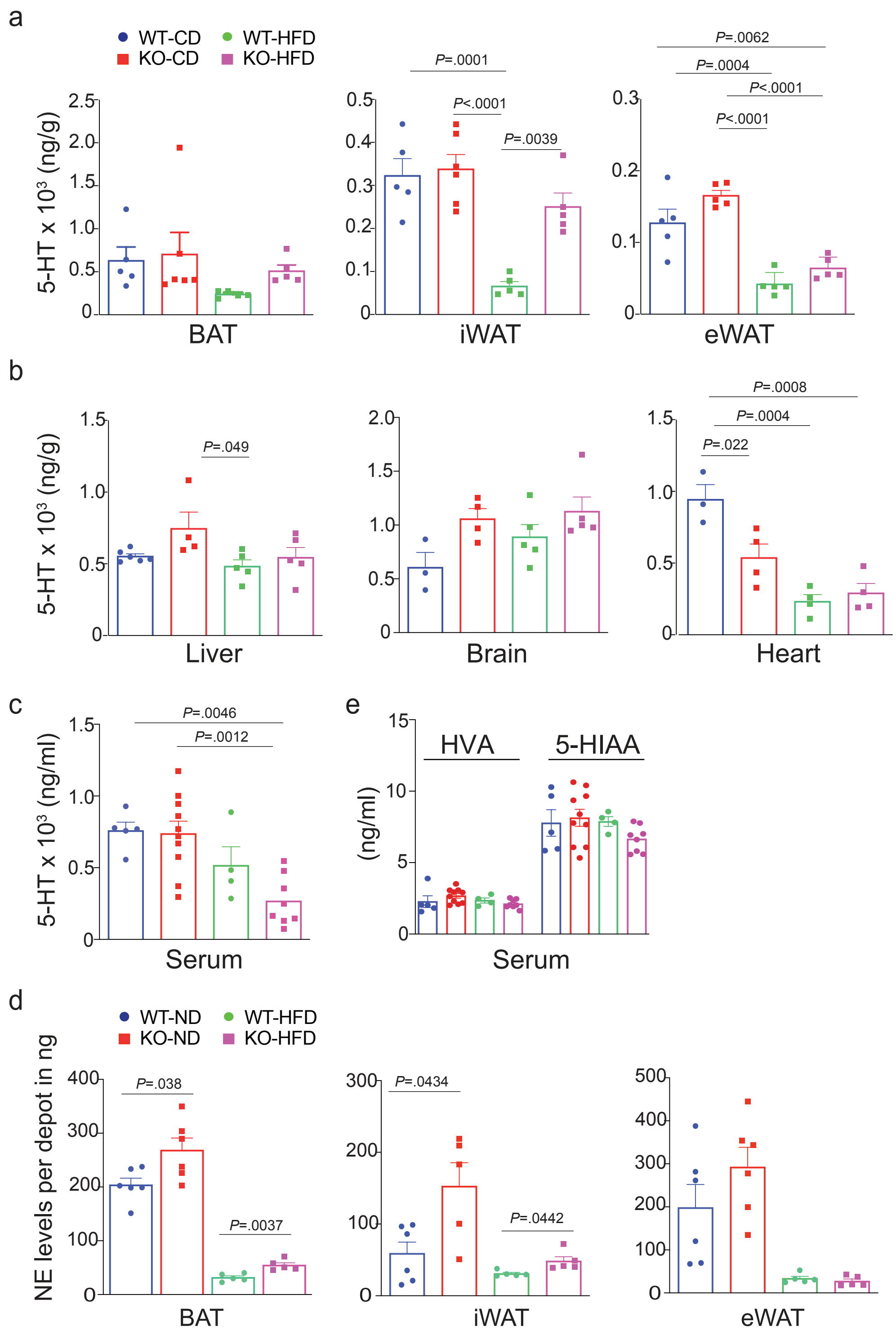

Supplementary Fig. 6. AIF1 deficiency does not suppress adipose tissue 5-HT levels, but increases NE adipose depot content of mice on CD and HFD. 5-HT and NE levels of control and AIF1-deficient mice fed with CD or HFD for 16 weeks were measured using LC/MS. **a**–**c** 5-HT levels in **a** adipose depots (n: BAT, WT-CD, 5; KO-CD, 6; iWAT and eWAT, WT-HFD, 5; KO-HFD, 5), **b** liver (n: WT-CD, 6; KO-CD, 4; WT-HFD, 5; KO-HFD, 5), brain (n: WT-CD, 3; KO-CD, 4; WT-HFD, 5; KO-HFD, 5) and heart (n: WT-CD, 3; KO-CD, 4; WT-HFD, 4; KO-HFD, 4), and **c** serum (n: WT-CD, 5; KO-CD, 10; WT-HFD, 4; KO-HFD, 8). **d** NE levels represented as ng/ per depot of tissue. (n: BAT, WT-CD, 6; KO-CD, 6; iWAT and eWAT, WT-HFD, 5; KO-HFD, 5). **e** serum homovanillic acid (HVA) and 5-Hydroxyindoleacetic acid (5-HIAA) in serum (n: BAT, WT-CD, 5; KO-CD, 10; iWAT WT-HFD, 4; KO-HFD, 8). Data are presented as mean ± s.e.m.; significance assessed by one-way ANOVA followed by Tukey's multiple comparison test. n indicates the number of biologically independent animals. Data are represen-tative of two similar experiments. Source data are provided as a Source Data file.

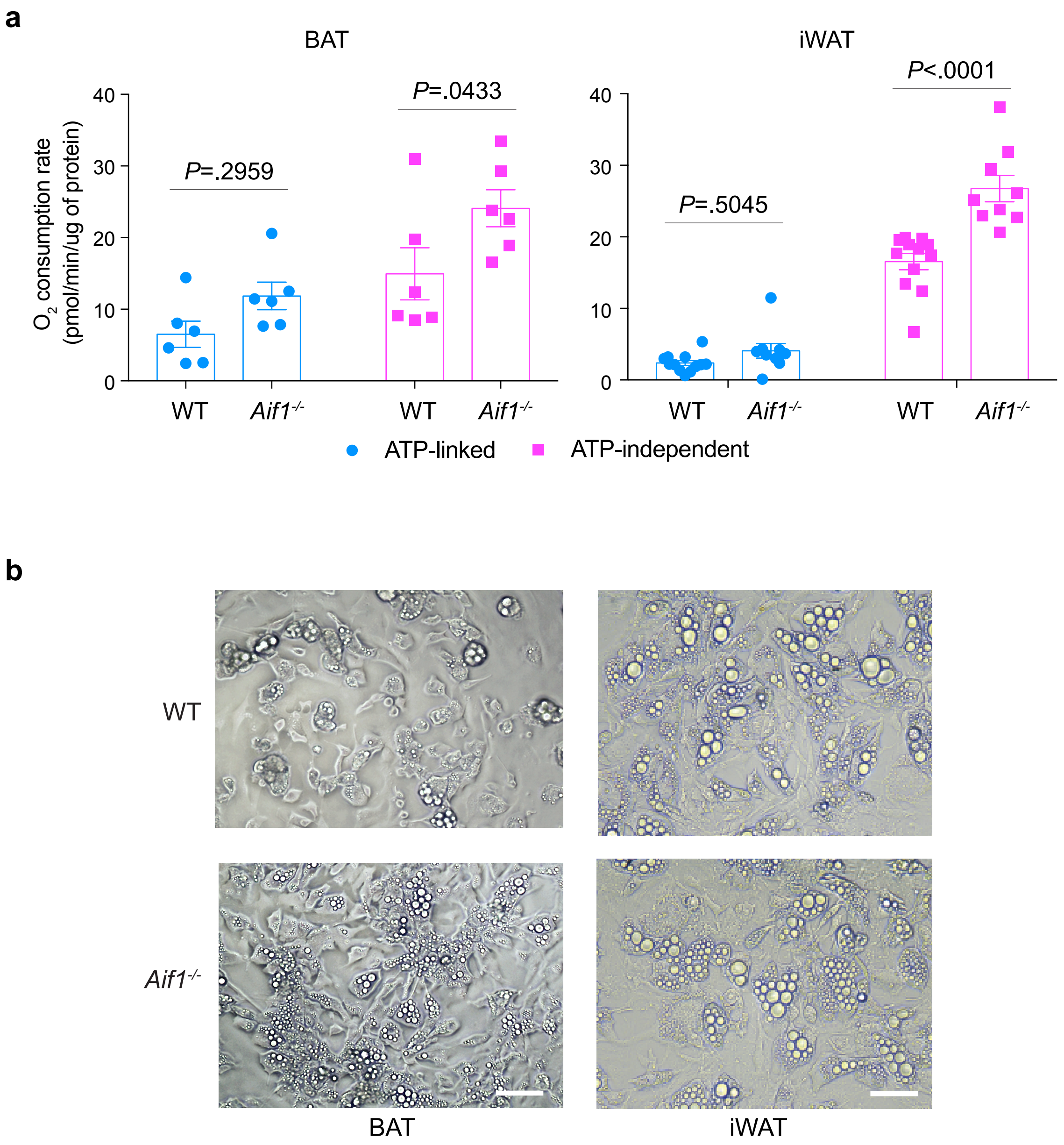

Supplementary Fig. 7. ATP-independent oxygen consumption is increased in *in vitro* differentiated AIF1-deficient adipocytes. Adipose depots were harvested from 6 (BAT) or 3 (iWAT) mice of each genotype, and pooled cells were differentiated *in vitro* toward adipo-cyte lineage (see Methods). Datapoints (n: (BAT, WT 6; *Aif1*$^{-/-}$ 6) and (iWAT, WT 12; *Aif1*$^{-/-}$ 9) represent independent cultures of the pooled preadipocytes. **a** Oxygen consumption prior to and after oligomycin treatment was measured using a Seahorse extracellular flux assay. The significance of observed differences was evaluated using Sidak's multiple compari-sons test. **b** Photomicrographs of differentiated WT and *Aif1*$^{-/-}$ cells derived from different depots, as indicated. Scale bars, 50 µm. Data are representative of 3 independent experi-ments. Source data are provided as a Source Data file.

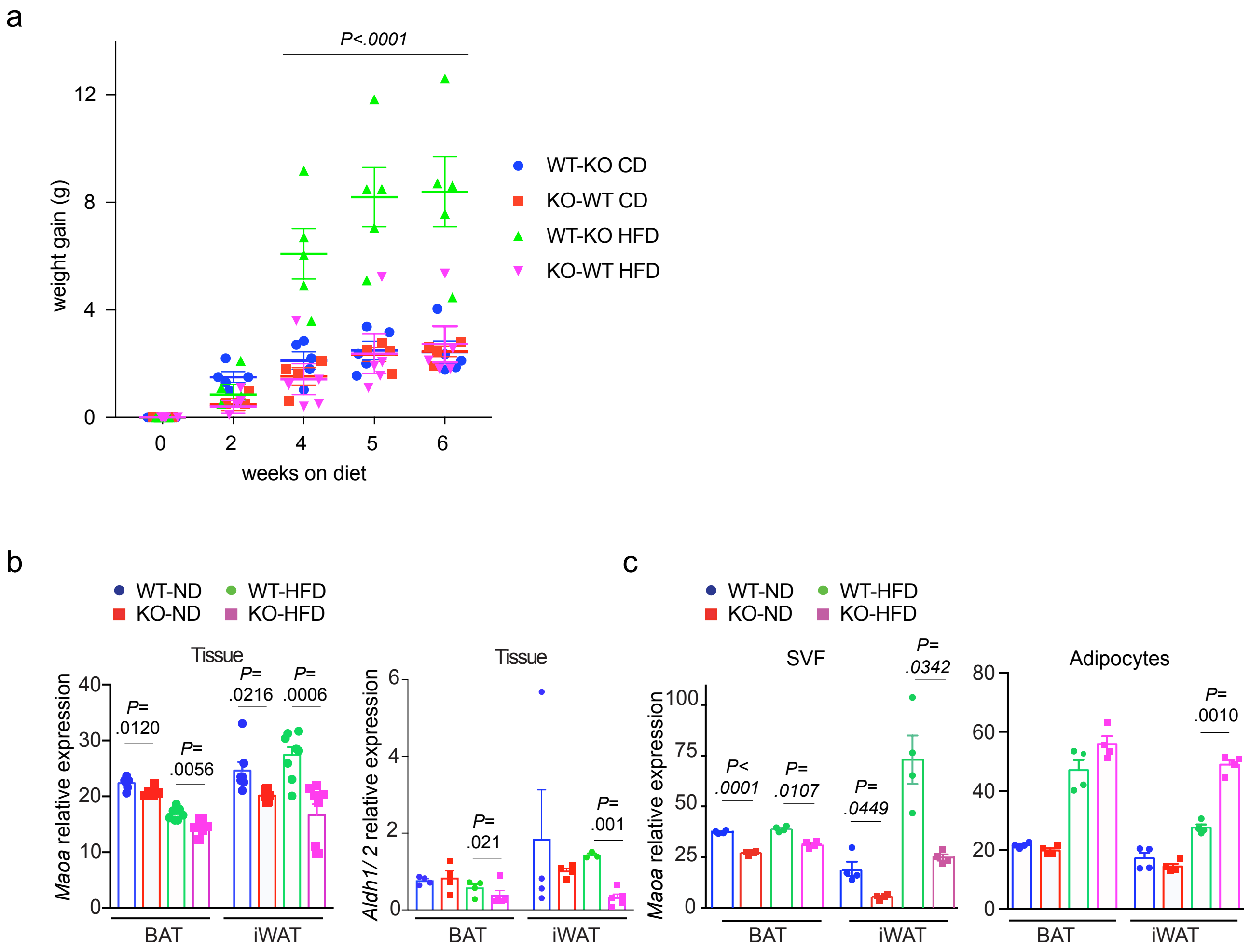

Supplementary Fig. 8. Role of AIF1 in bone marrow transplantation, obesity, and catecholamine catabolic enzyme expression in adipose tissues. **a** Total bone marrow cells from WT or *Aif1*$^{-/-}$ donor mice (age 6–8 weeks) were transferred to irradiated WT or *Aif1*$^{-/-}$ recipient mice (age 4–6 weeks). After 6 weeks of recovery, mice were randomly assigned to receive either CD or HFD for 6 weeks and weighed regularly. Key indicates donor-recipient diet, e.g., WT-KO CD. Graph shows weight gain over time (n: WT-KO CD, 5; KO-WT CD, 4; WT-KO HFD, 5; KO-WT HFD, 5). Data are presented as mean ± s.e.m. Significance assessed by two-way ANOVA with Tukey's multiple com-parisons test. P<.0001, WT-KO HFD vs other groups**. b, c** Expression of catecholamine catabolic enzyme transcripts in adipose tissues. **b** *Maoa* (n, BAT: WT-CD, 6; KO-CD, 6; WT-HFD, 7; KO-HFD, 7; n, iWAT: WT-CD, 7; KO-CD, 6; WT-HFD, 8; KO-HFD, 8) and *Aldh1/2* (n=4 per group) levels assessed by qRT-PCR in whole adipose tissues from WT or *Aif1*$^{-/-}$ mice. **c** *Maoa* levels evaluated by qRT-PCR in SVF and adipocytes separated from BAT and iWAT of WT and *Aif1*$^{-/-}$ mice fed with CD or HFD (n= 4 per group). Data in b and c are representative of two similar experiments and presented as mean ± s.e.m. Significance assessed by two-sided unpaired Student's t-test. n indicates number of biologically independent animals. Source data are provided as a Source Data file.

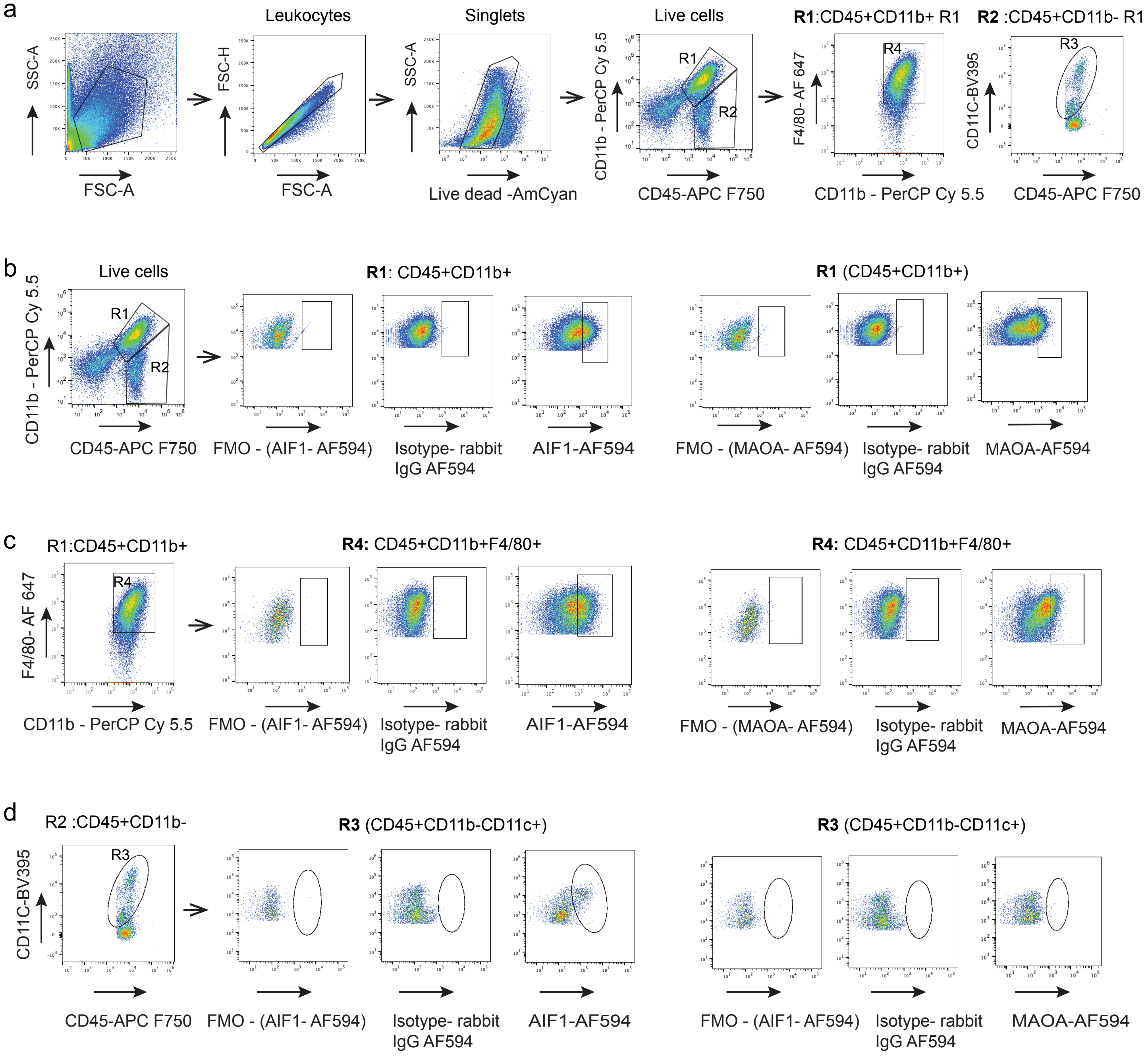

Supplementary Fig. 9. Gating strategy for AIF1 and MAOA expression on monocytes, DCs, and macrophages isolated from stromal vascular fractions of AT after 16 weeks on HFD. **a**, Gating strategy for monocyte, macrophages and dendritic cells in adipose tissue (AT) stromal vascular fractions. **b–d** Representative pseudoplot of flow cytometric analysis for AIF1 and MAOA expression with FMO (fluorescence mius other antibodies) and isotype control (rabbit IgG) on CD45+CD11b+ (AT-monocytes, **b**), CD45+CD11b+ F4/80+ (ATMs, **c**), CD45+CD11b-CD11c+ (AT-DCs, **d**) populations.

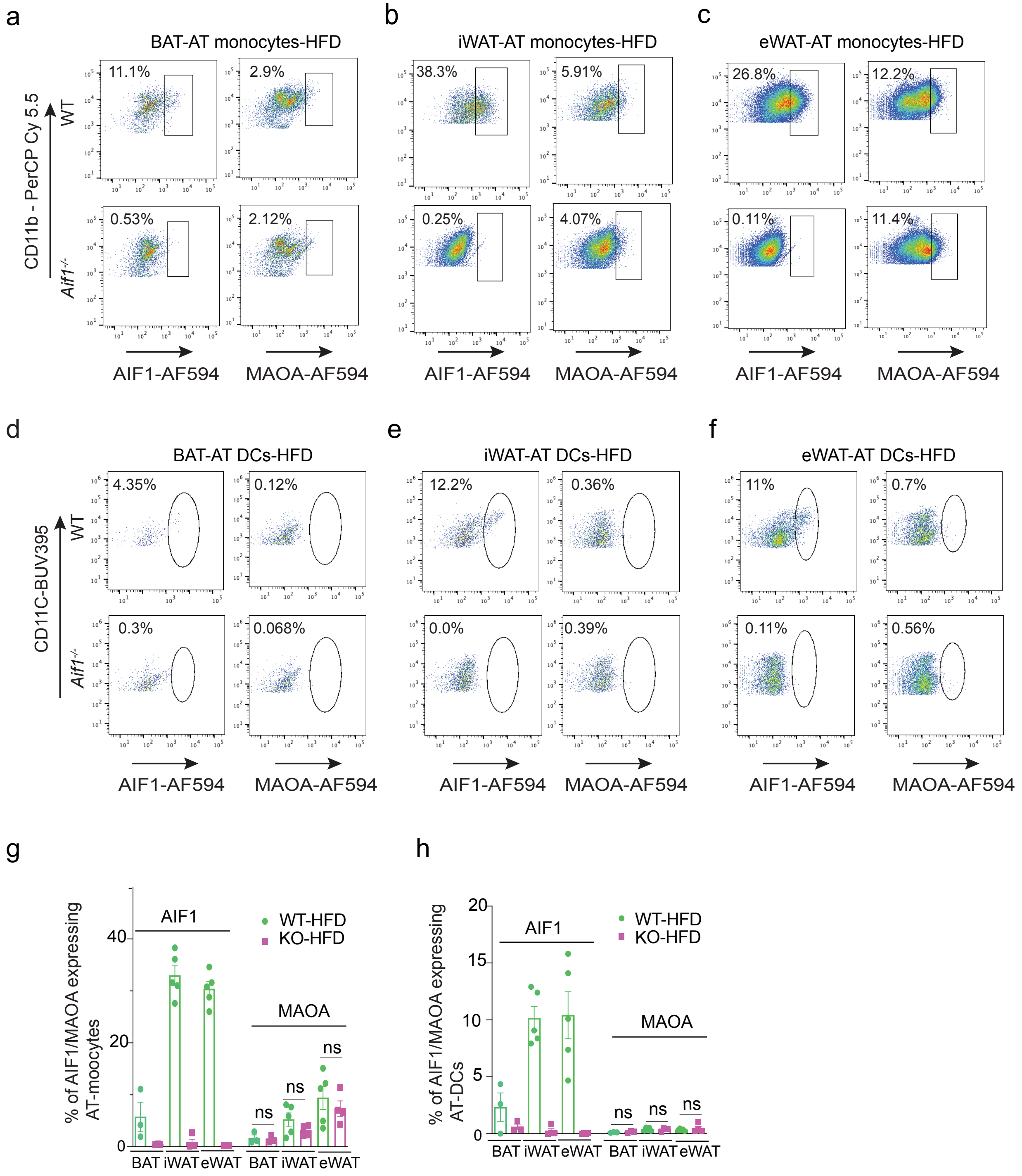

Supplementary Fig. 10. AIF1 is not required for catecholamine catabolic enzyme expression in adipose tissue (AT) monocytes and dendritic cells (DCs). AIF1 and MAOA expression on monocytes, DCs, and macrophages isolated from stromal vascular fractions of AT after 16 weeks on HFD. **a**–**c** and **g** Representative pseudoplot of flow cytometric analysis of AIF1 and MAOA expression in CD45+CD11b+ populations from stromal vascular fractions of BAT (**a**), iWAT (**b**), eWAT (**c**), and quantification expressed as % AIF1 and MAOA+ AT monocytes (**g**) (n, BAT: WT-HFD, 3; KO-HFD, 3; n, iWAT, eWAT: WT-HFD, 5; KO-HFD, 4). **d**–**f** and **h** Representative pseudoplots of flow cytometric analysis of AIF1 and MAOA expression in CD45+CD11b-CD11c+ populations from stromal vascular fractions of BAT (**d**), iWAT (**e**), eWAT (**f**) and quantification expressed as % AIF1 and MAOA+ of AT DCs (**h**) (n, BAT: WT-HFD, 3; KO-HFD, 3; n, iWAT, eWAT: WT-HFD, 5; KO-HFD, 4). Data are presented as mean ± s.e.m., with significance assessed by two-way ANOVA followed by Sidak’s multiple comparisons test. Data are representative of two similar experiments, and n indicates the number of biologically independent animals. Source data are provided as a Source Data file.

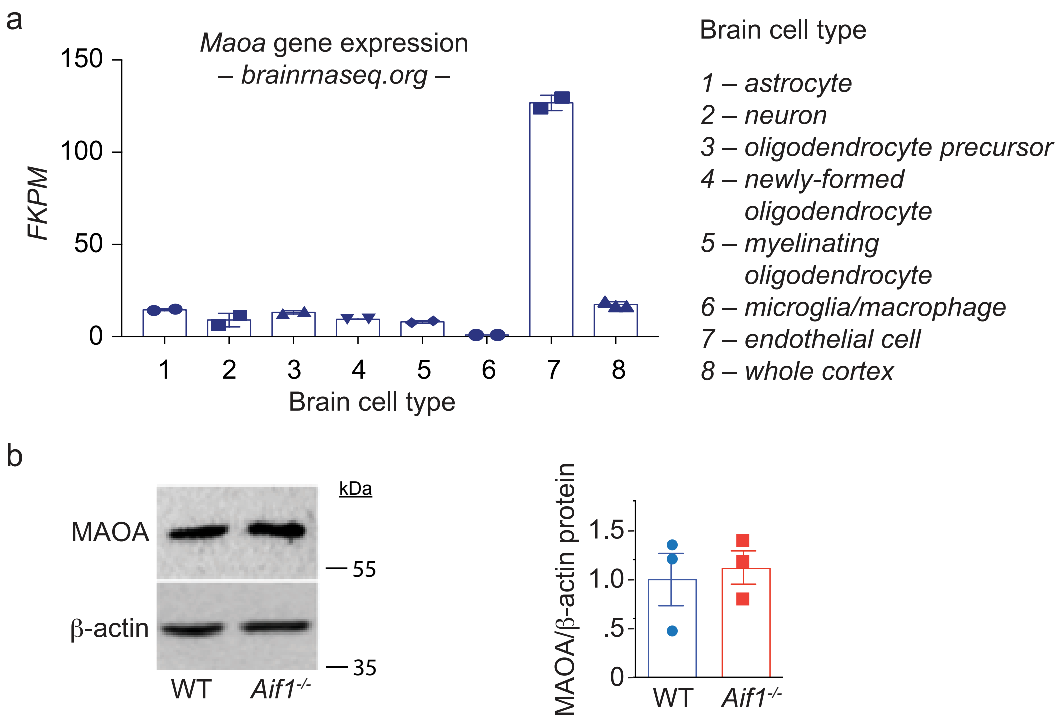

Supplementary Fig. 11. Loss of AIF1 does not change MaoA levels in the mouse brain. **a** Expression of *Maoa* transcripts (in FKPM) in different mouse brain cell types evaluated by RNA-seq. Data recovered from the brainrnaseq.org resource[41], with 2 biologically independent replicates per cell type. Each replicate is derived from pooled cortices from 3–12 animals[42]. **b** MAOA protein levels in mouse brain relative to β-actin, assessed by immunoblotting. Left panel shows representative blot image, while right panel shows quantitative analysis from n = 3 mice per genotype, normalized to WT level. For **a, b,** data are presented as mean ± s.e.m. Source data are provided as a Source Data file. FKPM, fragments per kilobase of exon per million mapped reads.

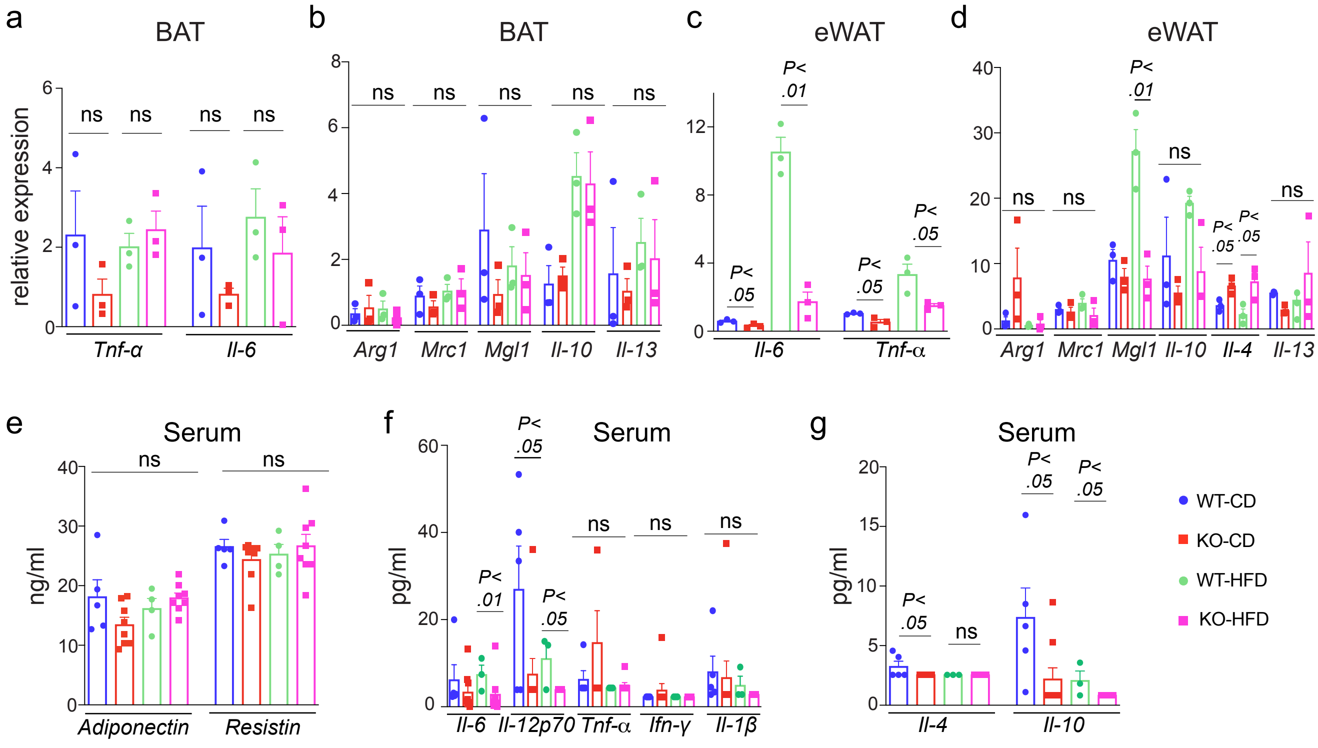

Supplementary Fig. 12. Loss of AIF1 promotes M2 vs. M1 cytokine gene expression only in eWAT. **a**–**d** Relative gene expression of pro-inflammatory cytokines in BAT (**a**) and eWAT (**c**); relative anti-inflammatory gene expression in BAT (**b**) and eWAT (**d**) (n=3 per group) evaluated on CD or HFD after 16 weeks using qRT-PCR, with normalization to β-actin. **e**–**g** Cytokine levels in serum measured after 16 weeks on CD and HFD using Magnetic Luminex assay. For **e**, n: WT-CD, 5; KO-CD, 8; WT-HFD, 4; KO-HFD, 8. For **f, g,** n: WT-CD, 5; KO-CD, 9; WT-HFD, 3; KO-HFD, 8). Data are presented as mean ± s.e.m., with significance assessed by unpaired two-sided Student's t-test. Data are representative of two similar experiments; n indicates the number of biologically independent animals. Source data are provided as a Source Data file.

Supplementary Tables

Supplementary Table 1	Gene expression by qRT-PCR			
	BAT		iWAT	
**Samples**	***Ucp1***	***Actb***	***Ucp1***	***Actb***
	C_T	C_T	C_T	C_T
WT-CD	19.71	21.12	31.16	20.49
WT-CD	19.28	20.87	32.05	20.78
WT-CD	18.98	20.81	29.70	20.59
WT-CD	19.51	21.09	31.67	20.58
KO-CD	18.08	21.50	31.79	23.01
KO-CD	17.65	20.92	30.54	22.24
KO-CD	18.58	21.10	30.58	22.45
KO-CD	17.56	21.05	30.61	20.82
WT-HFD	17.92	20.77	30.27	20.56
WT-HFD	17.20	20.66	30.35	20.77
WT-HFD	18.17	20.59	30.41	21.82
WT-HFD	17.26	20.50	29.49	20.79
KO-HFD	17.06	21.65	29.50	21.23
KO-HFD	17.66	22.09	32.09	22.28
KO-HFD	17.13	22.06	31.02	21.19
KO-HFD	18.13	22.95	27.50	25.00
KO-HFD	17.56	22.07	30.75	22.34

Supplementary Table 2 | Clinical materials

Deidentified sample	Procedure	Diabetes Status	BMI	Infectious Hx
1	Panniculectomy	No	31.1	None
2	Dog Ear Excision	No, Pre-DM2 prior to bariatric surgery	32.7	None
3	Panniculectomy	No	33.1	None
4	Panniculectomy	No	29	None
5	Panniculectomy	No	30.3	None
6	Panniculectomy	No	28.5	None
7	Dog Ear Excision	No	34.3	None
8	Panniculectomy	No, DM2 prior to bariatric surgery	31.6	None
9	Panniculectomy	No, DM2 prior to bariatric surgery	31.6	None
10	Panniculectomy	Pre-DM2	29.2	None
11	Panniculectomy	No, Pre-DM2 before bariatric surgery	28.3	None

Discarded tissues were obtained from 1 male and 10 female subjects ranging in age from 36–61

Supplementary Table 3 | **Antibodies and suppliers**

Antibody	Company	Catalog #	References
AIF1	Abcam	ab178847	https://labchemwako.fujifilm.com/us/product/detail/W01W0101-2000.html
AIF1	Wakofujifilm	016-20001	https://www.abcam.com/iba1-antibody-epr16589-ab178847.html;
MAOA	Abcam	ab126751	https://www.abcam.com/monoamine-oxidase-amao-a-antibody-epr7101-ab126751.html
UCP1	Thermofisher	PA1-24894	https://www.thermofisher.com/antibody/product/UCP1-Antibody-Polyclonal/PA1-24894
P-HSL	Cell Signaling	4126	https://www.cellsignal.com/products/primary-antibodies/phospho-hsl-ser660-antibody/4126
T-HSL	Cell Signaling	4107	https://www.cellsignal.com/products/primary-antibodies/hsl-antibody/4107?site-search-type=Products
OXPHOS	Abcam	Ab110413	https://www.abcam.com/total-oxphos-rodent-wb-antibody-cocktail-ab110413.html
β-actin	Abcam	ab8226	https://www.abcam.com/beta-actin-antibody-mabcam-8226-loading-control-ab8226.html
Chrome pure Rabbit IgG	Jackson ImmunoReserach Laboratories Inc	111-035-144	https://www.jacksonimmuno.com/catalog/products/111-035-144
Donkey-anti-rabbit IgG 594	ThermoFisher Life technologies	A-21207	https://www.thermofisher.com/antibody/product/Donkey-anti-Rabbit-IgG-H-L-Secondary-Antibody-Polyclonal/R37119
Mouse BD Fc Block (rat anti-mouse CD16/CD32	BD Biosciences	553142, clone:2.4G2	http://www.bdbiosciences.com/us/applications/research/b-cell-research/surface-markers/mouse/purified-rat-anti-mouse-cd16cd32-mouse-bd-fc-block-24g2/p/553142
Zombie Yellow	Biolegend	423103	https://www.biolegend.com/en-us/products/zombie-yellow-fixable-viability-kit-8514.

CD45 - APC/Fire 750	Biolegend	103153,<br><br>clone:30F11	https://www.biolegend.com/en-us/products/apc-fire-750-anti-mouse-cd45-antibody-13049
F4/80-A647	Biorad (AbD Serotec),	MCA497A647, clone: A3-1)	https://www.bio-rad-antibodies.com/monoclonal/mouse-f4-80-antibody-cl-a3-1-mca497.html?f=alexa%20fluor%C2%AE%20647
CD11B-PE	BD Biosciences	561689, clone:M1/70	http://www.bdbiosciences.com/us/applications/research/stem-cell-research/mesenchymal-stem-cell-markers-bone-marrow/mouse/negative-markers/pe-rat-anti-cd11b-m170/p/561689
CD11C-BUV395	BD Biosciences	564080, clone: HL3	https://www.bdbiosciences.com/us/reagents/research/antibodies-buffers/immunology-reagents/anti-mouse-antibodies/cell-surface-antigens/buv395-hamster-anti-mouse-cd11c-hl3/p/564080
CD11B-FITC	Biolegend	101206, clone:M1/70	https://www.biolegend.com/en-us/products/fitc-anti-mousehuman-cd11b-antibody-347
Goat-anti-rabbit IgG-horseradish peroxidase	Jackson ImmunoResearch Laboratories	111-035-144	https://www.jacksonimmuno.com/catalog/products/111-035-144

Supplementary Table 4 | Primers for RT-PCR

**Transcript** (m-mouse; h-human)	**Primer Sequence**
Aif1-m	F:5’-GGATTTGCAGGGAGGAAAAG-3’<br>R:5’-TGGGATCATCGAGGAATTG-3’
Ucp1-m	F: 5’-GGCCTCTACGACTCAGTCCA-3’<br>R: 5’-TAAGCCGGCTGAGATCTTGT-3’
Elovl3-m	F:5’-ACTTCGAGACGTTTCAGGACTTA-3’<br>R: 5’-GACGACCACTATGAGAAATGAGC-3’
Elovl6-m	F: 5’-CAGCAAAGCACCCGAACTA-3’<br>R: 5’-AGGAGCACAGTGATGTGGTG-3’
Dio2-m	F:5’-CTGCGCTGTGTCTGGAAC-3’<br>R: 5’-GGAGCATCTTCACCCAGTTT-3’
Cidea-m	F: 5’AAACCATGACCGAAGTAGCC-3’<br>R: 5’-AGGCCAGTTGTGATGACTAAGAC-3’
Tmem26-m	F:5’-CTGCTCAACCTCTTGCTGGT-3’<br>R: 5’-AAGATGGCCGGAGAAAGC-3’
Eva1-m	F:5’-TGTGCTTCCACTTCTCCTGA-3’<br>R:5’-TCCACAGCTTCTGTAGGACAAA-3’
Tbx1-m	F:5’-TTTGTGCCCGTAGATGACAA-3’<br>R: 5’-CTCGGCCAGGTGTAGCAG-3’
Fbxo31-m	F: 5’-AACCAGACAGATCACCACAGG3’<br>R: 5’-CAGCCACGTCCGAAACTC-3’
Ebf3-m	F: 5’-GCACAACAATTCCAAACACG-3’<br>R: 5’-CTTGATACACGGAGTGGCTTC-3’
Maoa-m	F: 5’-CGGATATTCTCAGTCACCAATG-3’<br>R: 5’-ATTTGGCCAGAGCCACCTA-3’
Aldh1l1-m	F: 5’-AATGACAAGGTGCCAGGTG-3’<br>R: 5’-TCGAGTTGAAAAACGTCAGC-3’
Aldh1l2-m	F: 5’-CGCTCGCTCCTCTACATCAT-3’<br>R: 5’-GCCAACTTCAGCTTGTTTTTG-3’
β -actin-m	F: 5’-CTAAGGCCAACCGTGAAAAG-3’<br>R: 5’-ACCAGAGGCATACAGGGACA-3’
AIF1-h	F: 5’-GGATTTACAGGGAGGAAAAGC-3’<br>R: 5’-TGGGATCGTCTAGGAATTG-3’
MAOA-h	F:5’-GGCTGCTACACGGCCTACT-3’<br>R:5’-ACGGGTTGACGAATCACC-3’
ALDH1L2-h	F: 5’-TCATTTATCACCCATCCATCCT-3’<br>R: 5’-CCCAGCTTTCTTATCTCCCATA-3’
β-ACTIN-h	F: 5’-TCCAAATATGAGATGCGTTGTT-3’<br>F: 5’-TGCTATCACCTCCCCTGTGT-3’
