# Supplementary material for: Increased adipose catecholamine levels and protection from obesity with loss of Allograft Inflammatory Factor-1
Source: Nat Commun. 2023 Jan 3;14:38. doi: 10.1038/s41467-022-35683-7 (PMC9810600; doi:10.1038/s41467-022-35683-7)
Supplement: Supplementary file 2 — Description of Additional Supplementary Files [file 41467_2022_35683_MOESM2_ESM.pdf]

Description of Additional Supplementary Files

File Name: Supplementary Data 1
Description: Pathway analysis (Ingenuity pathway analysis of RNA-seq dataset from wt and Aif1-/- bone marrow macrophages)
