# Supplementary material for: Increased adipose catecholamine levels and protection from obesity with loss of Allograft Inflammatory Factor-1
Source: Nat Commun. 2023 Jan 3;14:38. doi: 10.1038/s41467-022-35683-7 (PMC9810600; doi:10.1038/s41467-022-35683-7)
Supplement: Supplementary file 4 — Reporting Summary [file 41467_2022_35683_MOESM4_ESM.pdf]

nature portfolio

Corresponding author(s): Nicholas Sibinga

Last updated by author(s): Nov 1, 2022

Reporting Summary

Nature Portfolio wishes to improve the reproducibility of the work that we publish. This form provides structure for consistency and transparency in reporting. For further information on Nature Portfolio policies, see our Editorial Policies and the Editorial Policy Checklist.

Statistics

For all statistical analyses, confirm that the following items are present in the figure legend, table legend, main text, or Methods section.

n/a	Confirmed	
☐	☒	The exact sample size (*n*) for each experimental group/condition, given as a discrete number and unit of measurement
☐	☒	A statement on whether measurements were taken from distinct samples or whether the same sample was measured repeatedly
☐	☒	The statistical test(s) used AND whether they are one- or two-sided<br>*Only common tests should be described solely by name; describe more complex techniques in the Methods section.*
☐	☒	A description of all covariates tested
☐	☒	A description of any assumptions or corrections, such as tests of normality and adjustment for multiple comparisons
☐	☒	A full description of the statistical parameters including central tendency (e.g. means) or other basic estimates (e.g. regression coefficient) AND variation (e.g. standard deviation) or associated estimates of uncertainty (e.g. confidence intervals)
☐	☒	For null hypothesis testing, the test statistic (e.g. *F*, *t*, *r*) with confidence intervals, effect sizes, degrees of freedom and *P* value noted<br>*Give P values as exact values whenever suitable.*
☒	☐	For Bayesian analysis, information on the choice of priors and Markov chain Monte Carlo settings
☒	☐	For hierarchical and complex designs, identification of the appropriate level for tests and full reporting of outcomes
☐	☒	Estimates of effect sizes (e.g. Cohen's *d*, Pearson's *r*), indicating how they were calculated

Our web collection on statistics for biologists contains articles on many of the points above.

Software and code

Policy information about availability of computer code

Data collection	Images were captured using a Zeiss Axio imager. Whole body fat and lean masses were assessed by MRI (Echo-MRI). Whole body scanning was evaluated by computerized tomography scanning (La Theta CT, Hitachi Aloka). Gene expression levels were quantified by real time PCR using a Light Cycler (Roche) or ViiA 7 cycler system (Applied Biosystems). RNA-seq data were acquired on the Illumina Platform NEXTSeq500 using NSQ® 500 hi-Output KT v2. Flow data was collected by BD FACS Diva 4.0 software. Western blots images were acquired using an Odyssey Clx scanner (Li-Cor). Enzyme activity and ELISA data were acquired with a Varioskan LUX multimode Reader (ThermoFisher)..

Data analysis

RNA-seq: FastQC files from RNA-seq experiments were trimmed of their adapters with trim_galore (v0.5.0), fastqc (v0.11.5) and cutadapt (v1.15) under default parameters. Reads were aligned to the GRCm38 mouse genome with Tophat (v2.0.13) (Trapnell, C et al., TopHat: discovering splice junctions with RNA-Seq. Bioinformatics 25, 1105–1111 (2009)) with parameters allowing a read to be mapped to at most one location: "--no-coverage-search -p 1 -g 1". Gene hits were counted with HTseq (v0.6.1) (Anders, S et al., HTSeq--a Python framework to work with high throughput sequencing data. Bioinformatics 31, 166–169 (2015)) under default parameters using release 84 of the Mus_musculus, GRCm38 GTF annotation file [(https://www.ncbi.nlm.nih.gov/assembly/GCF_000001635.20/)].
Differential expression analysis was performed in R/Bioconductor following the DESeq2 workflow as described (Love, M et al.,. Moderated estimation of fold change and dispersion for RNA-seq data with DESeq2. Genome Biol. 15, 550 (2014)) and annotated with biomaRt (Durinck, S., et al., Mapping identifiers for the integration of genomic datasets with the R/Bioconductor package biomaRt. Nat.Protocols 4, 1184–1191 (2009)).
Pathway analysis for the RNA-seq data set was performed using Ingenuity software v. 01-14 (Qiagen Bioinformatics).
Flow cytometry: Flow data analyses were performed using Flow Jo software package v. 10.5.3 (Tree Star).
Immunofluorescence, immunohistochemistry analysis, and Western blot quantitation were performed using ImageJ bundled with Java 8 (open source NIH software). Western blot quantitation was performed by Image studio lite version 5.2.5 (LI-COR).
Graphing and statistical analysis were performed with GraphPad Prism v. 7.0. Figures were assembled using Adobe Illustrator v. 23.0.

For manuscripts utilizing custom algorithms or software that are central to the research but not yet described in published literature, software must be made available to editors and reviewers. We strongly encourage code deposition in a community repository (e.g. GitHub). See the Nature Portfolio guidelines for submitting code & software for further information.

Data

Policy information about availability of data

All manuscripts must include a data availability statement. This statement should provide the following information, where applicable:

- Accession codes, unique identifiers, or web links for publicly available datasets
- A description of any restrictions on data availability
- For clinical datasets or third party data, please ensure that the statement adheres to our policy

Data availability: All sequencing data that support the findings of this study have been deposited in the National Center for Biotechnology Information Gene Expression Omnibus (GEO) and are accessible through the GEO Series accession number GSE133278 [https://www.ncbi.nlm.nih.gov/geo/query/acc.cgi?acc=GSE133278].The reference mouse genome utilized can be found at Mus_musculus, GRCm38 GTF annotation file [(https://www.ncbi.nlm.nih.gov/assembly/GCF_000001635.20/)]. The metabolic and other gene expression data generated in this study are provided in the Supplementary Information or Source Data files, both of which are provided with this paper. Some of the raw human subjects data (beyond what is shown) are protected and not available due to data privacy considerations.

Human research participants

Policy information about studies involving human research participants and Sex and Gender in Research.

Reporting on sex and gender

This study reports on gene expression analysis of discarded tissues derived from 11 subjects undergoing liporeduction/cosmetic surgery. Sex or gender were not considered during collection of these samples, but skewed heavily toward female vs male subjects (10 vs. 1, respectively) based on self-reporting. Age and sex characteristics of this cohort are reported in aggregate to protect individual privacy, and are reported in Extended Data Table 3. The associated data are plotted together in Figure 5i. Because of the small number of male samples, separate analyses are not possible, but the single male sample is within the 95% confidence bounds of the overall relationship.

Population characteristics

Participants were candidates for liporeduction/cosmetic surgery. Information on covariates (Procedure, Diabetes Status, BMI, Infectious Hx) is provided in Extended Data Table 3. Sex and age are described in aggregate to protect individual privacy.

Recruitment

Subjects were not recruited for this study. Rather, the study materials were discarded tissues from subjects who underwent liporeduction/cosmetic surgery. There may be self-selection biases in this clinical population based on perceived body image and financial resources, but these are unlikely to affect the gene expression analyses performed for this study.

Ethics oversight

The relevant institution for these clinically-derived samples, New York University, allows for the collection of de-identified tissues for non-human subjects research based on PI self-certification, without a specific IRB number.

Note that full information on the approval of the study protocol must also be provided in the manuscript.

Field-specific reporting

Please select the one below that is the best fit for your research. If you are not sure, read the appropriate sections before making your selection.

☒ Life sciences ☐ Behavioural & social sciences ☐ Ecological, evolutionary & environmental sciences

For a reference copy of the document with all sections, see nature.com/documents/nr-reporting-summary-flat.pdf

Life sciences study design

All studies must disclose on these points even when the disclosure is negative.

Sample size	Sample sizes are noted in the Figure legends and in methods. Sample size in experiments involving mice was estimated based on the results of previous experiments (as optimized for in vivo evaluation). In some cases, an on-line calculator (http://homepage.stat.uiowa.edu/~rlenth/Power/old_index.html) was used to predict a sample size with a power of 80% to detect a difference in assessed effects at a level of significance of 0.05, and sample sizes were also informed by ethical considerations to restrict the number of animals. For some experiments, tissue samples were collected from individual mice or pooled from multiple mice as necessary to obtain sufficient material to perform the assays. For example, in flow cytometry and sorting experiments, tissues from 2–3 mice were pooled to yield sufficient stromal vascular cells and the corresponding CD45+CD11b+F4/80+ macrophage subset to perform AIF1 and MAOA expression analysis.
Data exclusions	For bone marrow transplantation studies, mice showing signs of poor engraftment after irradiation (less than 75% donor reconstitution of peripheral blood cells) were excluded from analysis.
Replication	Numbers of experimental repetitions are indicated in Figure legends and/or Methods. Repeatability and accuracy of results was supported by performing experiments with three independent biological replicates and validating results with multiple methods (e.g. metabolic phenotype and activity data, enzyme expression (RNA and protein) and activity, plus substrates; multiple cohorts of mice). Findings were successfully reproduced by more than one person in independent replicates.
Randomization	All animals and derived samples were randomly grouped based on the genotype.
Blinding	Blinding methods were used for assessing metabolic characteristics and adipose tissue characteristics for each genotype/mouse model used in this study, including analyses performed through core facilities. For other experiments involving quantitative (i.e., machine-derived) assessments, investigators were generally not blinded to the groups.

Reporting for specific materials, systems and methods

We require information from authors about some types of materials, experimental systems and methods used in many studies. Here, indicate whether each material, system or method listed is relevant to your study. If you are not sure if a list item applies to your research, read the appropriate section before selecting a response.

Materials & experimental systems

n/a	Involved in the study	
☐	☒	Antibodies
☐	☒	Eukaryotic cell lines
☒	☐	Palaeontology and archaeology
☐	☒	Animals and other organisms
☒	☐	Clinical data
☒	☐	Dual use research of concern

Methods

n/a	Involved in the study	
☒	☐	ChIP-seq
☐	☒	Flow cytometry
☒	☐	MRI-based neuroimaging

Antibodies

Antibodies used	AIF1 (Wako, cat #016-20001); 1:500 dilution)<br>AIF1 (Abcam, cat #ab178847; 1:500 dilution)<br>MAOA (Abcam, cat #ab126751; 1:2000 dilution)<br>UCP1 (Thermofisher, cat #PA1-24894; 1:500 dilution)<br>P-HSL (Cell Signaling, cat #4126; 1:500 dilution)<br>T-HSL Cell Signaling, cat #4107; 1:500 dilution)<br>β-actin (Abcam, cat #ab8226; 1:5000 dilution)<br>Chrome pure Rabbit IgG ((Jackson ImmunoResearch Laboratories, cat #011-000-003; 1 ug/ml)<br>Goat-anti-rabbit IgG- Horseradish peroxidase (Jackson ImmunoResearch Laboratories, cat #111-035-144; 1:2000 dilution)<br>Goat-anti-rabbit IgG 594 (ThermoFisher Life Technologies, cat #A-21207; 1:300 dilution)<br>Mouse BD Fc Block (rat anti-mouse CD16/CD32) (BD, cat #553142, clone: 2.4G2; 1:100 dilution)<br>Zombie Yellow (Biolegend, cat #423103; 1:100 dilution)<br>CD45 -APC/Fire 750 (Biolegend, cat #103153, clone: 30F11; 1:125 dilution)<br>F4/80- A647 (Biorad (AbD Serotec), cat #MCA497A647, clone: A3-1; 1:17 dilution)<br>CD11B -PE, (BD Biosciences, cat #561689, clone: M1/70; 1:200 dilution)<br>CD11c-BUV395, (BD Biosciences; 1:100 dilution)<br>anti-rabbit IRDye@800 CW (Li-COR; 1:5000 dilution)<br>anti-mouse-IRDye@680 CW (Li-COR; 1:5000 dilution)<br>Total OXPHOS Rodent WB Antibody Cocktail (Abcam, cat #ab110413; 1:150 dilution)
Validation	AIF1 (Wako, cat #016-20001) for western blot; validation: https://labchem wako.fujifilm.com/us/product/detail/W01W0101-2000.html; this manuscript Fig. 1

AIF1 (Abcam, cat #ab178847) for flow analysis; validation https://www.abcam.com/iba1-antibody-epr16589-ab178847.html; this manuscript Fig. 5
MAOA (Abcam, cat #ab126751); validation: https://www.abcam.com/monoamine-oxidase-amao-a-antibody-epr7101-ab126751.html
UCP1 (Thermofisher, cat #PA1-24894); validation: https://www.thermofisher.com/antibody/product/UCP1-Antibody-Polyclonal/PA1-24894
P-HSL (Cell Signaling, cat #4126); validation: https://www.cellsignal.com/products/primary-antibodies/phospho-hsl-ser660-antibody/4126
T-HSL Cell Signaling, cat #4107); validation: https://www.cellsignal.com/products/primary-antibodies/hsl-antibody/4107?site-search-type=Products
β-actin (Abcam, cat #ab8226); validation: https://www.abcam.com/beta-actin-antibody-mabcam-8226-loading-control-ab8226.html
Chrome pure Rabbit IgG ((Jackson ImmunoResearch Laboratories, cat #011-000-003); validation: https://www.jacksonimmuno.com/catalog/products/011-000-003
Goat-anti-rabbit IgG- Horseradish peroxidase (Jackson ImmunoResearch Laboratories, cat #111-035-144); validation: https://www.jacksonimmuno.com/catalog/products/111-035-144
Goat-anti-rabbit IgG 594 (ThermoFisher Life Technologies, cat #A-21207); validation: https://www.thermofisher.com/antibody/product/Donkey-anti-Rabbit-IgG-H-L-Secondary-Antibody-Polyclonal/R37119
Mouse BD Fc Block (rat anti-mouse CD16/CD32) (BD, cat #553142, clone: 2.4G2); validation: http://www.bdbiosciences.com/us/applications/research/b-cell-research/surface-markers/mouse/purified-rat-anti-mouse-cd16cd32-mouse-bd-fc-block-24g2/p/553142
Zombie Yellow (Biolegend, cat #423103); validation: https://www.biolegend.com/en-us/products/zombie-yellow-fixable-viability-kit-8514.
CD45 -APC/Fire 750 (Biolegend, cat #103153, clone: 30F11); validation: https://www.biolegend.com/en-us/products/apc-fire-750-anti-mouse-cd45-antibody-13049
F4/80- A647 (Biorad (AbD Serotec), cat #MCA497A647, clone: A3-1) validation: https://www.bio-rad-antibodies.com/monoclonal/mouse-f4-80-antibody-cl-a3-1-mca497.html?f=alexa%20fluor%C2%AE%20647
CD11B -PE, (BD, cat #561689, clone: M1/70); validation: http://www.bdbiosciences.com/us/applications/research/stem-cell-research/mesenchymal-stem-cell-markers-bone-marrow/mouse/negative-markers/pe-rat-anti-cd11b-m170/p/561689
Total OXPHOS Rodent WB Antibody Cocktail (Abcam, cat #ab110413; 1:150 dilution); validation: https://www.abcam.com/total-oxphos-rodent-wb-antibody-cocktail-ab110413.html#lb

Eukaryotic cell lines

Policy information about cell lines and Sex and Gender in Research

Cell line source(s): Cell line sources: We generated, immortalized, and maintained bone marrow-derived macrophages from 8 week old male WT and Aif1-/- mice using a validated protocol (Stanley, E. R. Murine bone marrow-derived macrophages. Methods Mol. Biol. 75, 301–304 (1997); Yu, W. et al. CSF-1 receptor structure/function in MacCsf1r-/- macrophages: regulation of proliferation, differentiation, and morphology. J. Leukoc. Biol. 84, 852–863 (2008)).

Authentication: Cells were validated by assessing AIF1 expression and F4/80 staining for macrophage identity using flow cytometry and Western blotting methods.

Mycoplasma contamination: Specific testing for mycoplasma was not performed

Commonly misidentified lines (See ICLAC register): No commonly misidentified cell lines were used in this study.

Animals and other research organisms

Policy information about studies involving animals; ARRIVE guidelines recommended for reporting animal research, and Sex and Gender in Research

Laboratory animals: Aif1-/- mice were backcrossed for at least 14 generations with mice of the C57BL/6J strain; WT C57BL/6J mice served as controls. Baseline characteristics have been previously reported (Casimiro, I et al., Genetic inactivation of the allograft inflammatory factor-1 locus. Genesis 51, 734–740 (2013)). Experimental conditions were started with 8 week old male mice, as mentioned in Methods. Housing conditions are also described in Methods, including light-dark cycle (12h/12h), temperature (22°C), and humidity (30–70%).

Wild animals: The study did not involve wild animals

Reporting on sex: The studies reported in this manuscript are of male mice only. The overall obesity phenotype is found both male and female mice, but the more in depth studies have been performed only in male mice to date. This is due in part to the smaller overall size of female mice, which carries over to smaller brown adipose tissue depots and lower cell yields that make flow cytometry and recovery of RNA from sorted cells more difficult.

Field-collected samples: The study did not involve field-collected samples

Ethics oversight: All animal experiments were conducted in accordance with NIH guidelines under protocols approved by the Institutional Animal Care and Use Committee of the Albert Einstein College of Medicine

Note that full information on the approval of the study protocol must also be provided in the manuscript.

Flow Cytometry

Plots

Confirm that:

☒ The axis labels state the marker and fluorochrome used (e.g. CD4-FITC).

☒ The axis scales are clearly visible. Include numbers along axes only for bottom left plot of group (a 'group' is an analysis of identical markers).

☒ All plots are contour plots with outliers or pseudocolor plots.

☒ A numerical value for number of cells or percentage (with statistics) is provided.

Methodology

Sample preparation	Adipose tissues from brown and white adipose depots were harvested, pooled from 2–3 mice per sample, and digested with Collagenase D (Roche) for 1h at 37°C with shaking. Cell suspensions were filtered through a 70 μm filter and centrifuged at 450g for 5 min. Single cell suspensions were lysed to remove red blood cells and stained for antibodies for flow cytometric analysis.
Instrument	Aria or LSRII (Becton Dickinson)
Software	Flow data were collected using BD FACS Diva™ software. Analyses were performed using FlowJo software.
Cell population abundance	Flow cytometry was used for quantitative and preparative analyses. We generally stain for 0.5–1 million cells obtained from tissues, and acquire 250, 000 events (equivalent to 250,000 cells). The total numbers for cells analyzed were 50,000 for BAT, 100,000 for iWAT and 250,000 for eWAT. During acquisition we examined 5000 to 10000 representative cells.
Gating strategy	All cells were gated for FSC-A/SSC-A plots to identify lymphocyte populations. Single cell population was achieved by gating on FSC-A/FSC-H, after which we eliminated dead cells by gating on the Zombie yellow negative population. In live cells, we gated on CD45+ cells, and then CD11b+F4-80+ double-positive cells were gated for either the AIF1 or MAOA population.

☒ Tick this box to confirm that a figure exemplifying the gating strategy is provided in the Supplementary Information.
